# Supplementary figures and images for: Uev1A counteracts oncogenic Ras stimuli in both polyploid and diploid cells
Source: eLife. 2026 Mar 25;14:RP107104. doi: 10.7554/eLife.107104 (PMC13016607; doi:10.7554/eLife.107104)

IP: HA

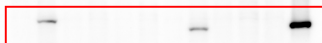

IB: Myc

Supplement: Figure 6—source data 1. [file elife-107104-fig6-data1.zip › Figure 6-source data 1. PDF file containing original western blots, indicating the relevant bands and treatments/Figure 6A-1.pdf]

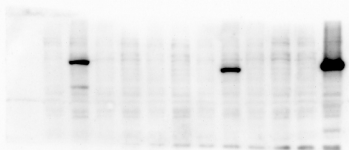

IP: HA

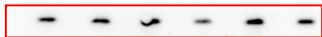

IB: HA

Supplement: Figure 6—source data 1. [file elife-107104-fig6-data1.zip › Figure 6-source data 1. PDF file containing original western blots, indicating the relevant bands and treatments/Figure 6A-2.pdf]

Input

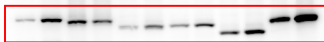

IB: Myc

Supplement: Figure 6—source data 1. [file elife-107104-fig6-data1.zip › Figure 6-source data 1. PDF file containing original western blots, indicating the relevant bands and treatments/Figure 6A-3.pdf]

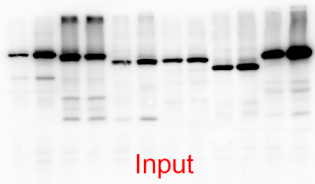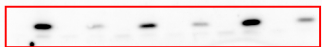

IB: HA

Supplement: Figure 6—source data 1. [file elife-107104-fig6-data1.zip › Figure 6-source data 1. PDF file containing original western blots, indicating the relevant bands and treatments/Figure 6A-4.pdf]

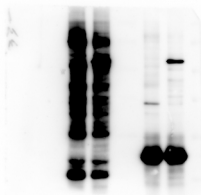

IP: Myc  
Input IP

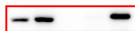

IB: Flag

Supplement: Figure 6—source data 1. [file elife-107104-fig6-data1.zip › Figure 6-source data 1. PDF file containing original western blots, indicating the relevant bands and treatments/Figure 6B-1.pdf]

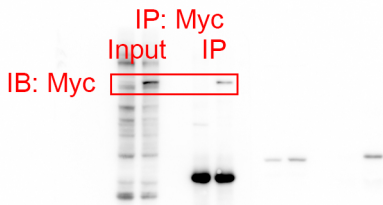

Supplement: Figure 6—source data 1. [file elife-107104-fig6-data1.zip › Figure 6-source data 1. PDF file containing original western blots, indicating the relevant bands and treatments/Figure 6B-2.pdf]

IP: Flag

Input

IP

IB: Myc

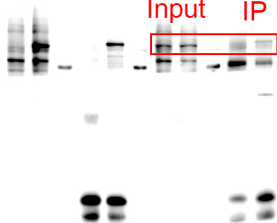

Supplement: Figure 6—source data 1. [file elife-107104-fig6-data1.zip › Figure 6-source data 1. PDF file containing original western blots, indicating the relevant bands and treatments/Figure 6B-3.pdf]

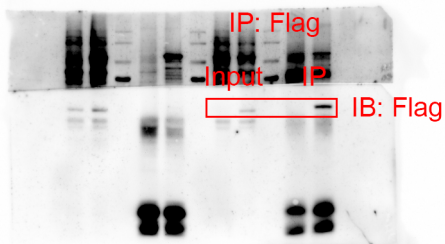

Supplement: Figure 6—source data 1. [file elife-107104-fig6-data1.zip › Figure 6-source data 1. PDF file containing original western blots, indicating the relevant bands and treatments/Figure 6B-4.pdf]

*GFP-RNAi*    *ben-RNAi*

IB: Flag-CycA

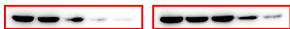

IB:  $\beta$ -Actin

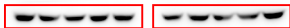

— — — — —      — — — — —

Supplement: Figure 6—source data 1. [file elife-107104-fig6-data1.zip › Figure 6-source data 1. PDF file containing original western blots, indicating the relevant bands and treatments/Figure 6C-1.pdf]

*uev1a-RNAi* *cdc27-RNAi*

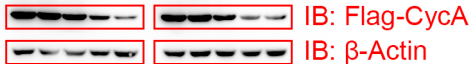

Supplement: Figure 6—source data 1. [file elife-107104-fig6-data1.zip › Figure 6-source data 1. PDF file containing original western blots, indicating the relevant bands and treatments/Figure 6C-2.pdf]

IB: Flag

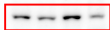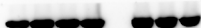

Supplement: Figure 6—source data 1. [file elife-107104-fig6-data1.zip › Figure 6-source data 1. PDF file containing original western blots, indicating the relevant bands and treatments/Figure 6E-1.pdf]

IB: Tubulin

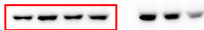

Supplement: Figure 6—source data 1. [file elife-107104-fig6-data1.zip › Figure 6-source data 1. PDF file containing original western blots, indicating the relevant bands and treatments/Figure 6E-2.pdf]

Input

IB: HA

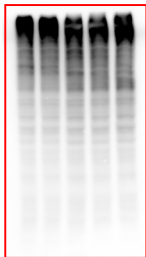

Supplement: Figure 6—source data 1. [file elife-107104-fig6-data1.zip › Figure 6-source data 1. PDF file containing original western blots, indicating the relevant bands and treatments/Figure 6F-1.pdf]

Input

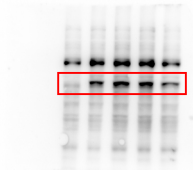

IB: Myc

Supplement: Figure 6—source data 1. [file elife-107104-fig6-data1.zip › Figure 6-source data 1. PDF file containing original western blots, indicating the relevant bands and treatments/Figure 6F-2.pdf]

Input

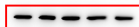

IB: Flag

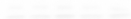

Supplement: Figure 6—source data 1. [file elife-107104-fig6-data1.zip › Figure 6-source data 1. PDF file containing original western blots, indicating the relevant bands and treatments/Figure 6F-3.pdf]

IP: Flag

IB: Flag

IB: HA

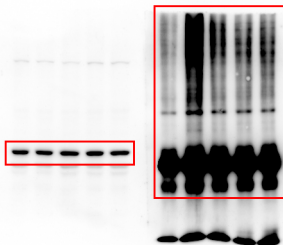

Supplement: Figure 6—source data 1. [file elife-107104-fig6-data1.zip › Figure 6-source data 1. PDF file containing original western blots, indicating the relevant bands and treatments/Figure 6F-4.pdf]

IP: Flag

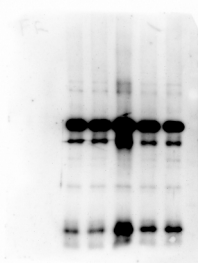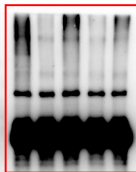

IB: HA

Supplement: Figure 6—source data 1. [file elife-107104-fig6-data1.zip › Figure 6-source data 1. PDF file containing original western blots, indicating the relevant bands and treatments/Figure 6G-1.pdf]

IP: Flag

IB: Flag

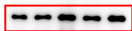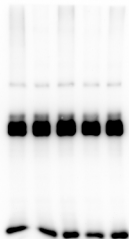

Supplement: Figure 6—source data 1. [file elife-107104-fig6-data1.zip › Figure 6-source data 1. PDF file containing original western blots, indicating the relevant bands and treatments/Figure 6G-2.pdf]

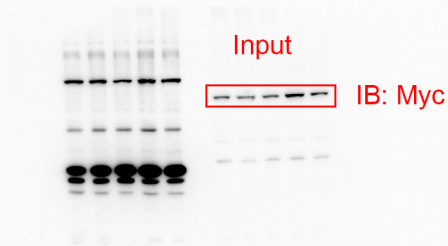

Supplement: Figure 6—source data 1. [file elife-107104-fig6-data1.zip › Figure 6-source data 1. PDF file containing original western blots, indicating the relevant bands and treatments/Figure 6G-3.pdf]

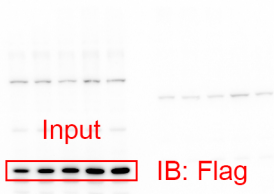

Supplement: Figure 6—source data 1. [file elife-107104-fig6-data1.zip › Figure 6-source data 1. PDF file containing original western blots, indicating the relevant bands and treatments/Figure 6G-4.pdf]

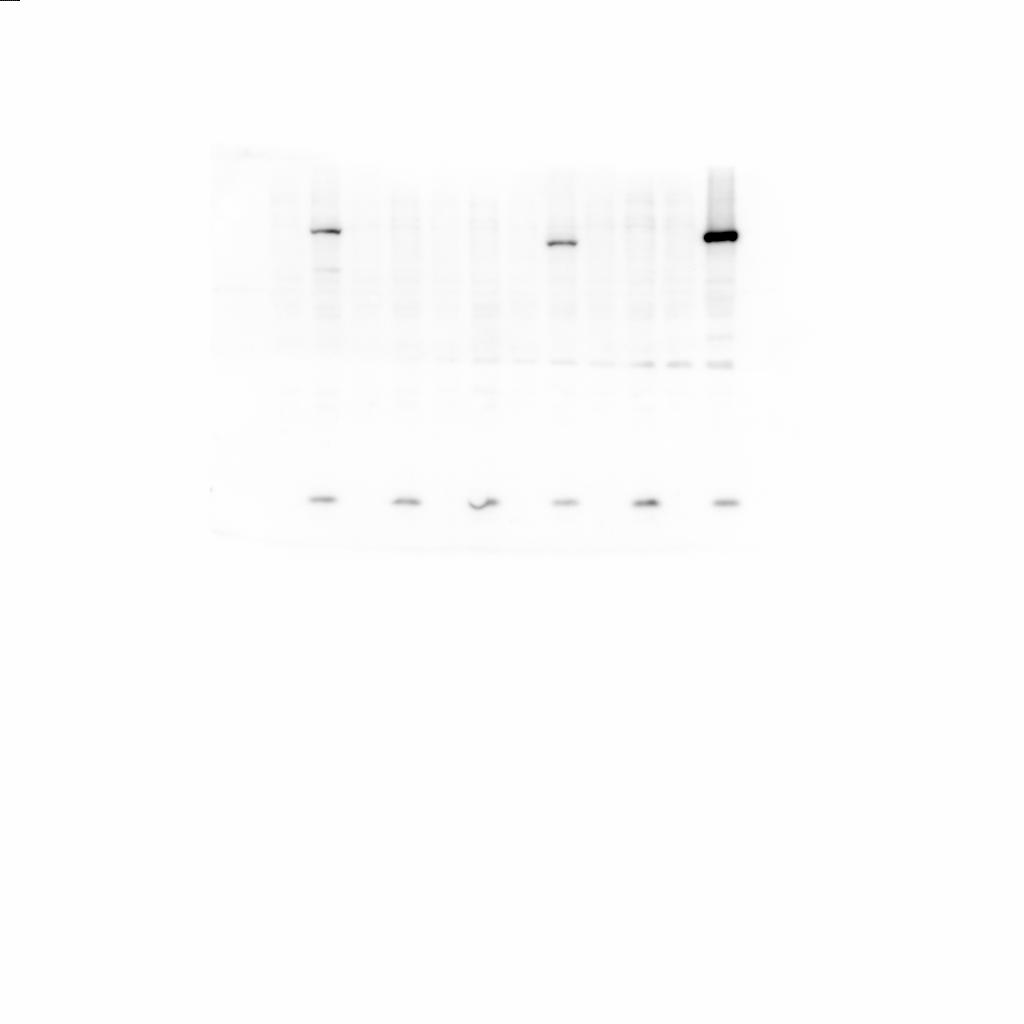

Supplement: Figure 6—source data 2. [file elife-107104-fig6-data2.zip › Figure 6-source data 2. Original files for western blot analysis/Figure 6A-1.TIF]

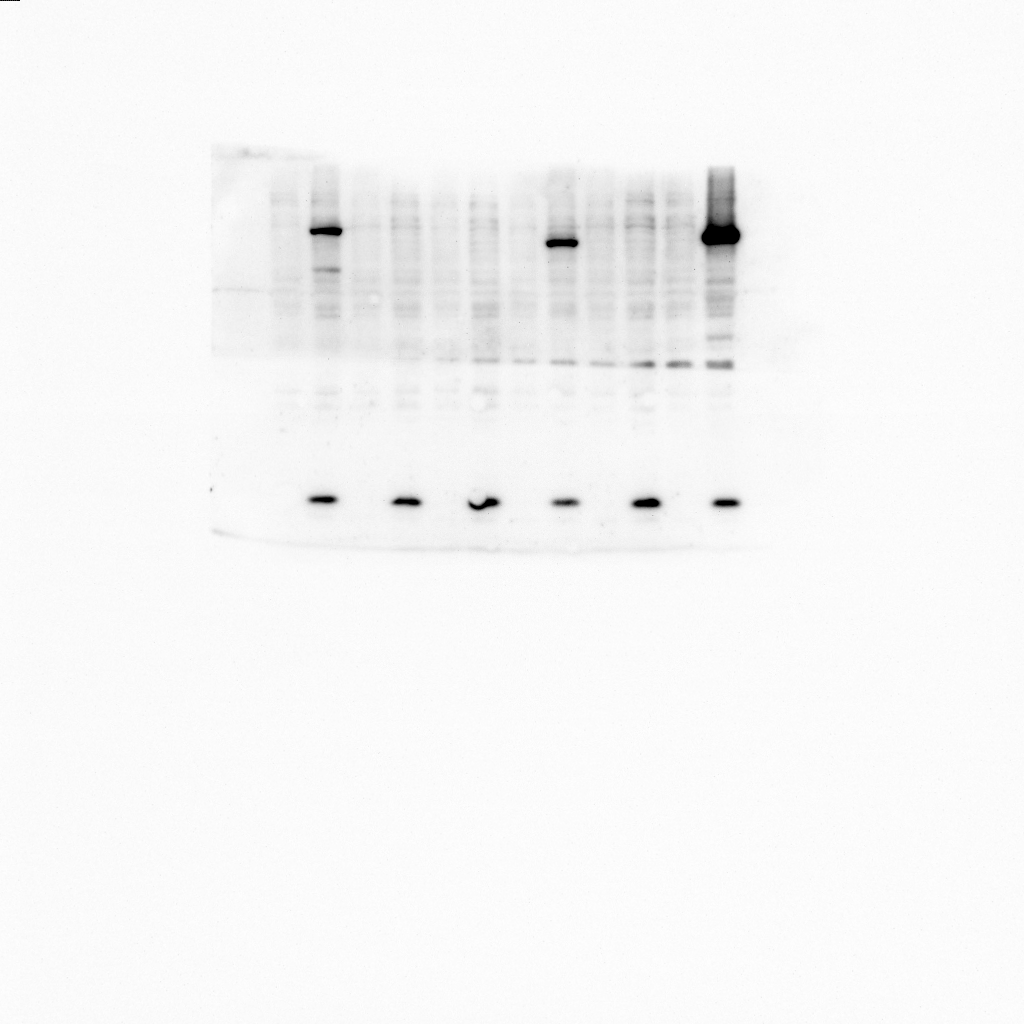

Supplement: Figure 6—source data 2. [file elife-107104-fig6-data2.zip › Figure 6-source data 2. Original files for western blot analysis/Figure 6A-2.TIF]

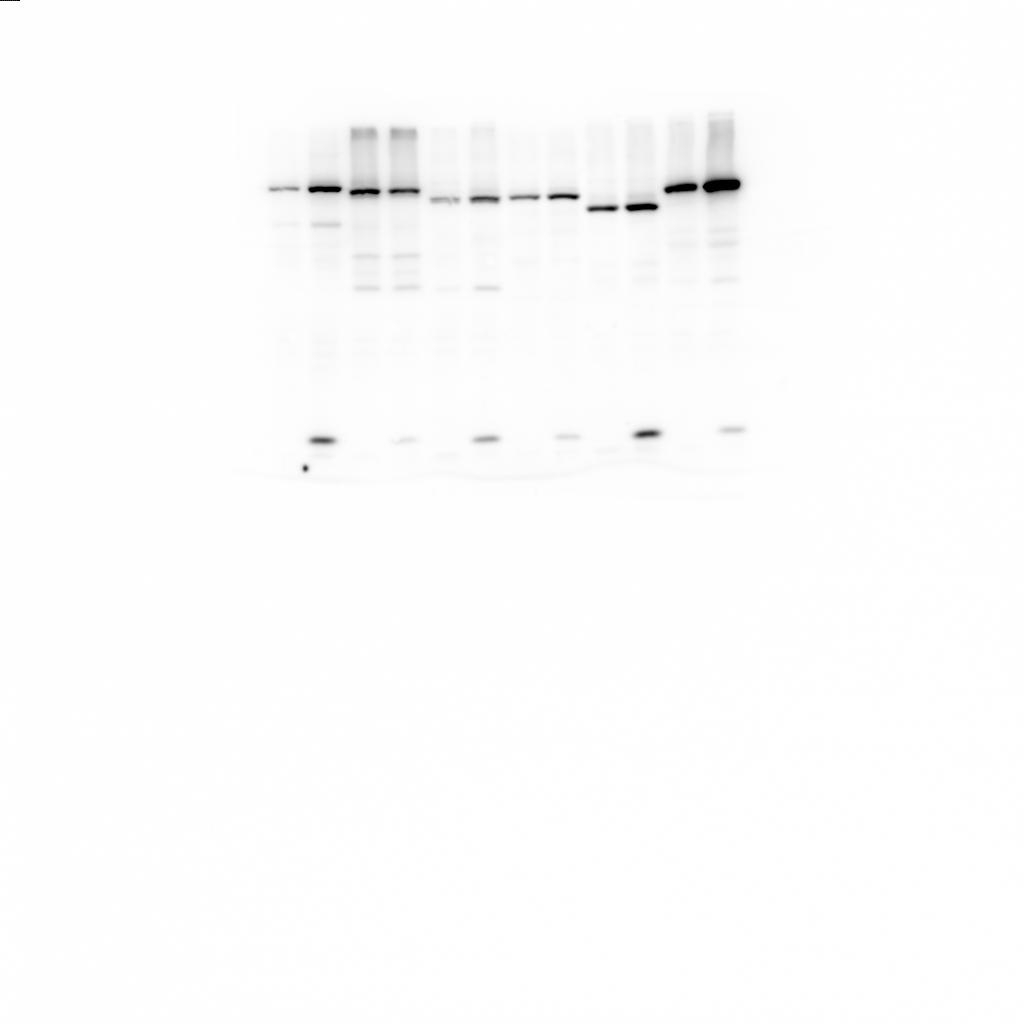

Supplement: Figure 6—source data 2. [file elife-107104-fig6-data2.zip › Figure 6-source data 2. Original files for western blot analysis/Figure 6A-3.tif]

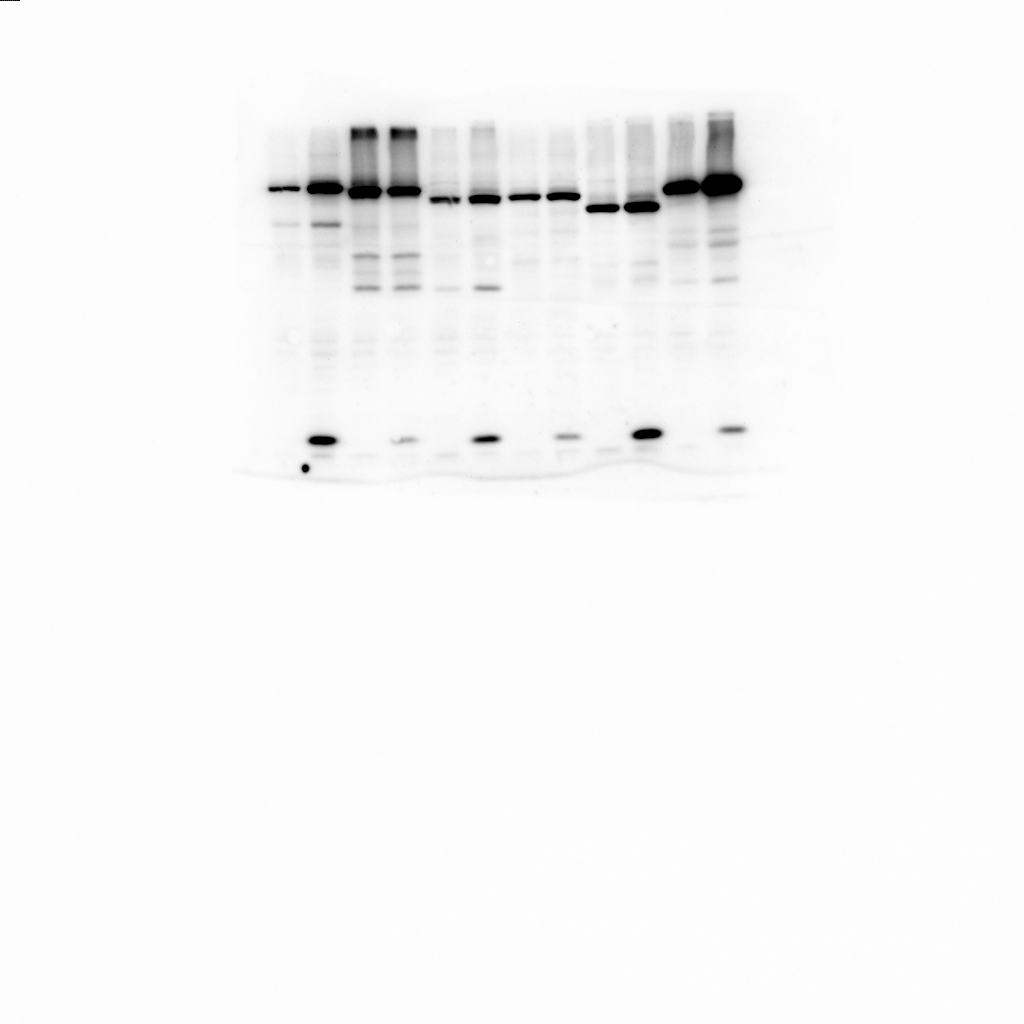

Supplement: Figure 6—source data 2. [file elife-107104-fig6-data2.zip › Figure 6-source data 2. Original files for western blot analysis/Figure 6A-4.tif]

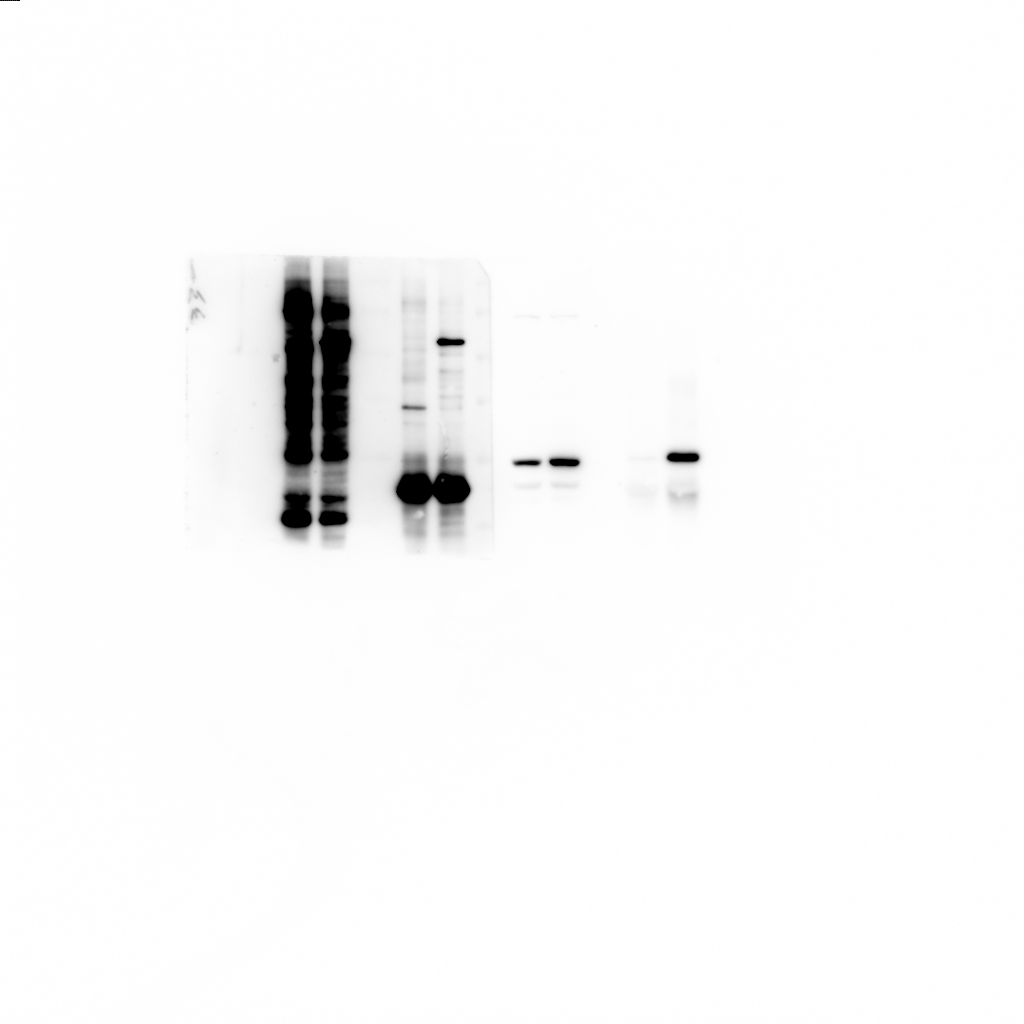

Supplement: Figure 6—source data 2. [file elife-107104-fig6-data2.zip › Figure 6-source data 2. Original files for western blot analysis/Figure 6B-1.TIF]

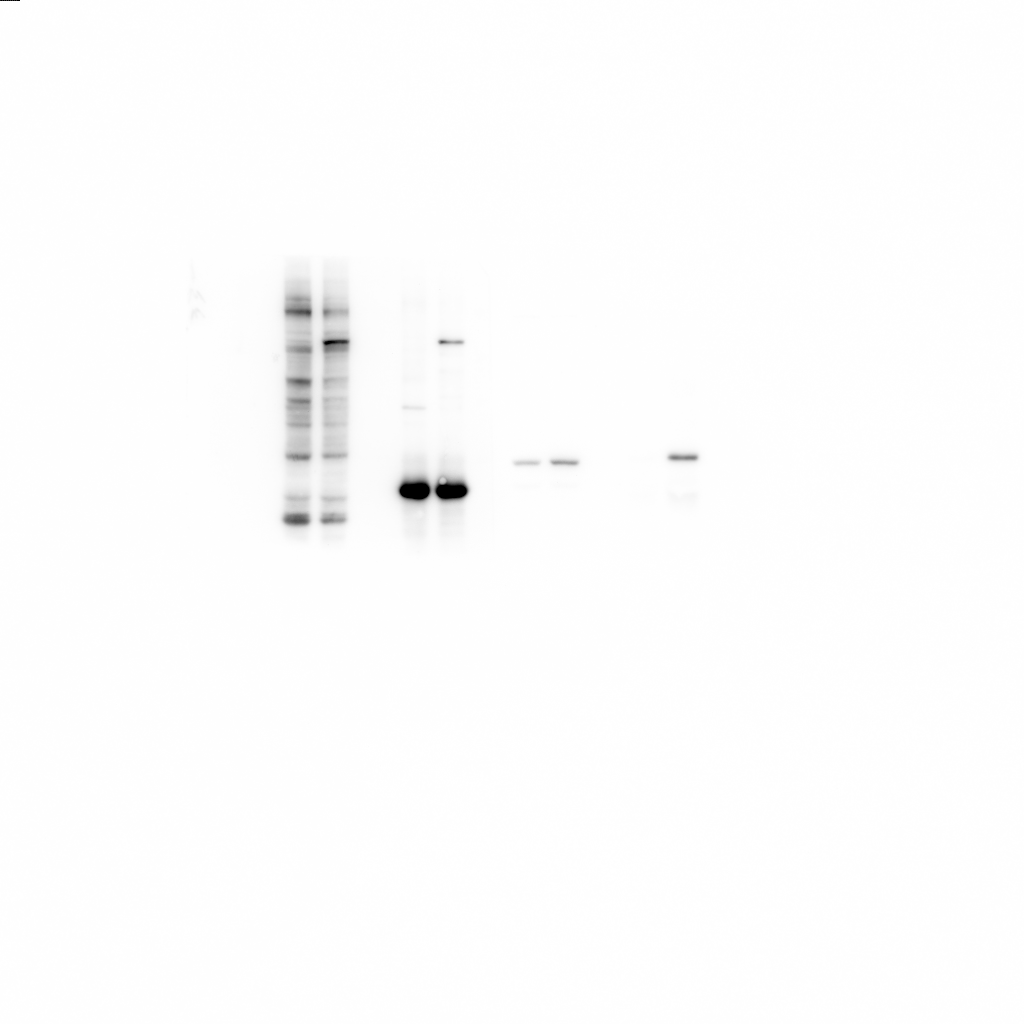

Supplement: Figure 6—source data 2. [file elife-107104-fig6-data2.zip › Figure 6-source data 2. Original files for western blot analysis/Figure 6B-2.TIF]

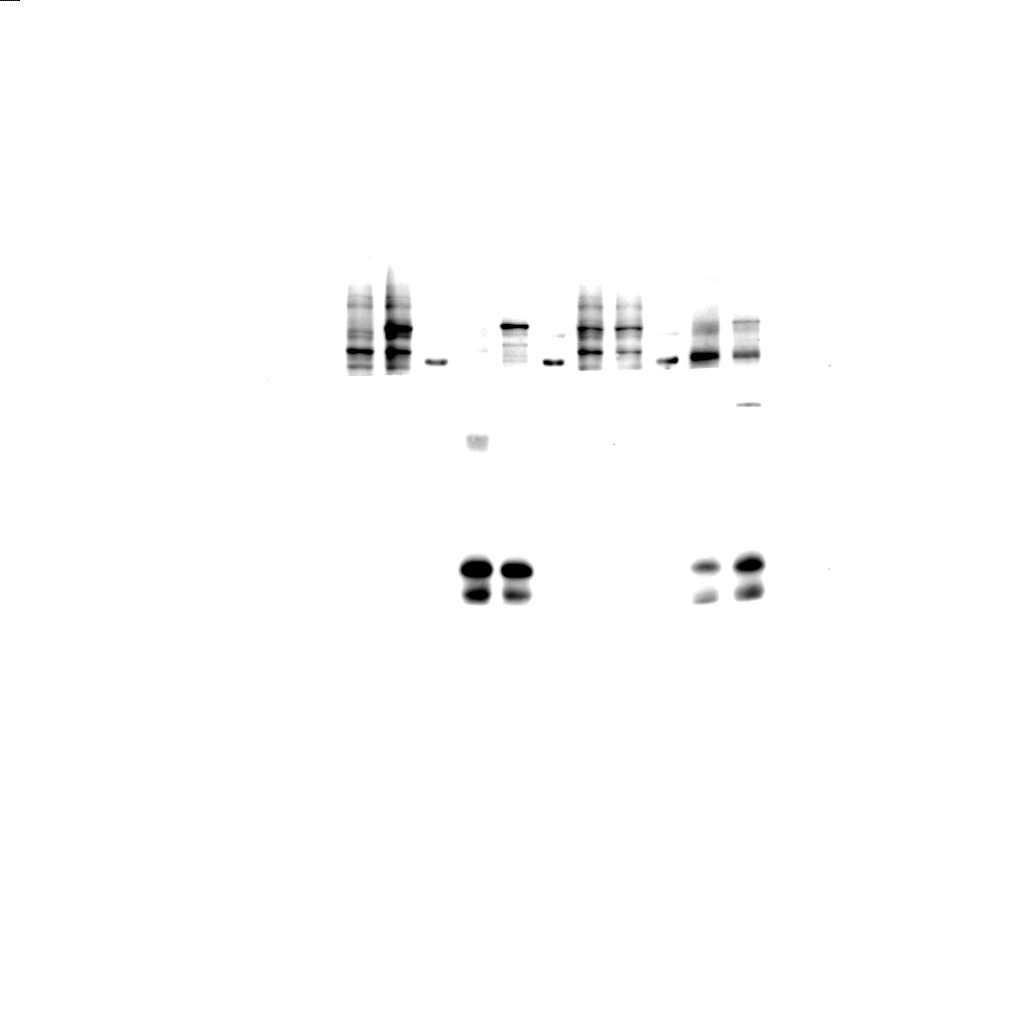

Supplement: Figure 6—source data 2. [file elife-107104-fig6-data2.zip › Figure 6-source data 2. Original files for western blot analysis/Figure 6B-3.TIF]

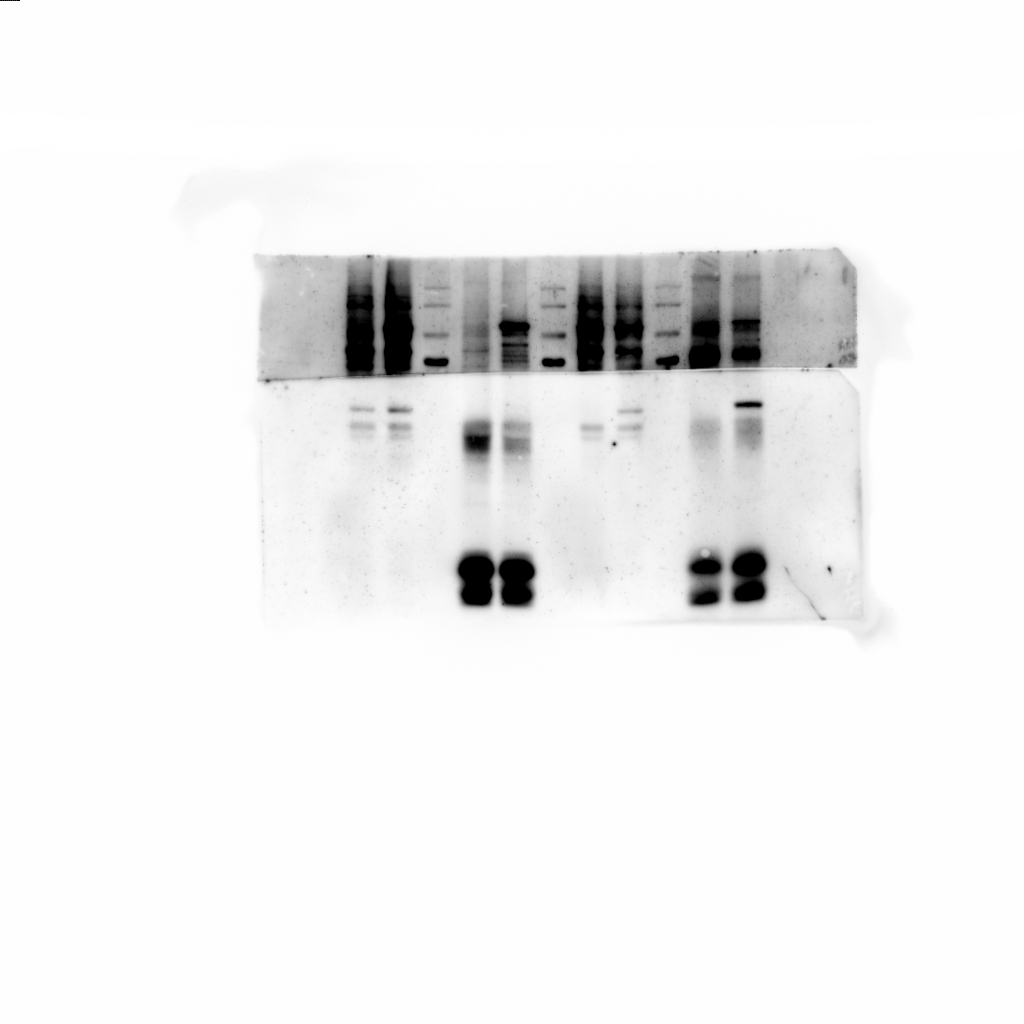

Supplement: Figure 6—source data 2. [file elife-107104-fig6-data2.zip › Figure 6-source data 2. Original files for western blot analysis/Figure 6B-4.TIF]

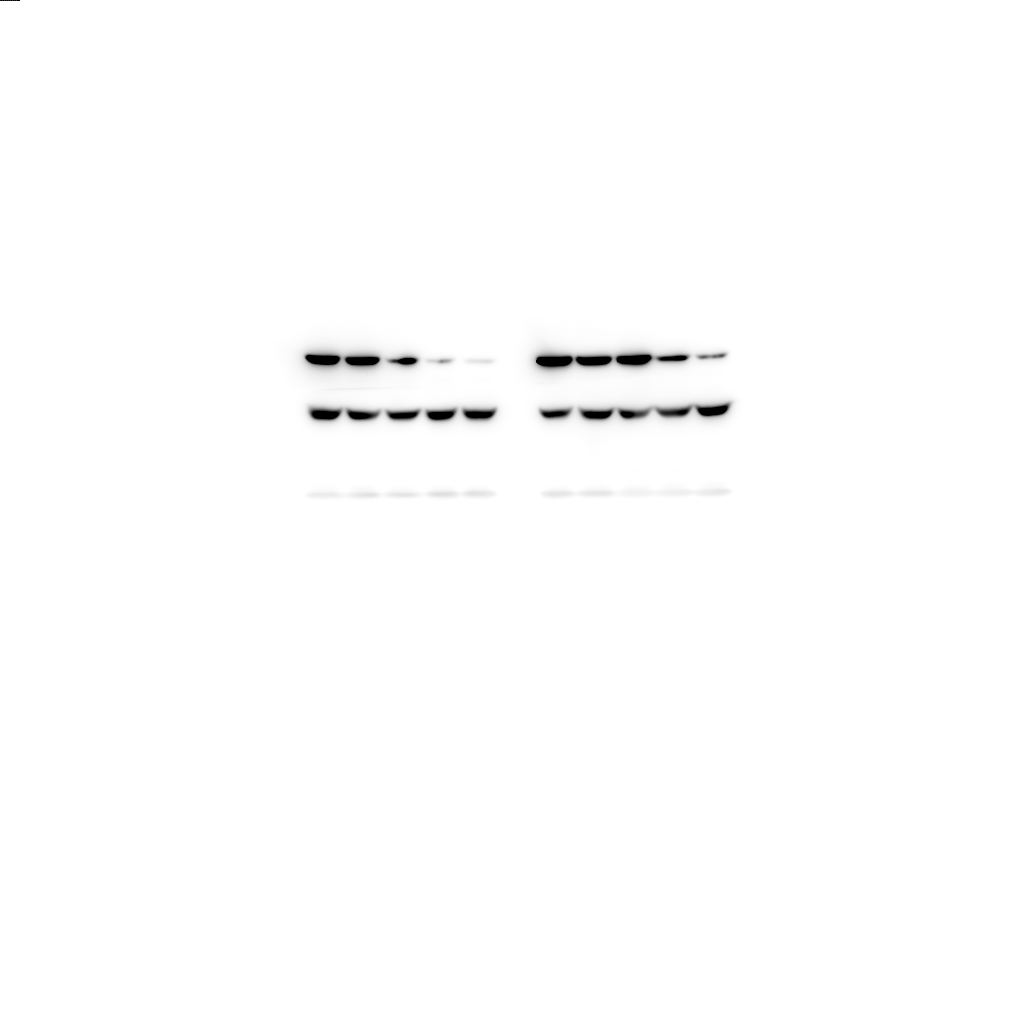

Supplement: Figure 6—source data 2. [file elife-107104-fig6-data2.zip › Figure 6-source data 2. Original files for western blot analysis/Figure 6C-1.TIF]

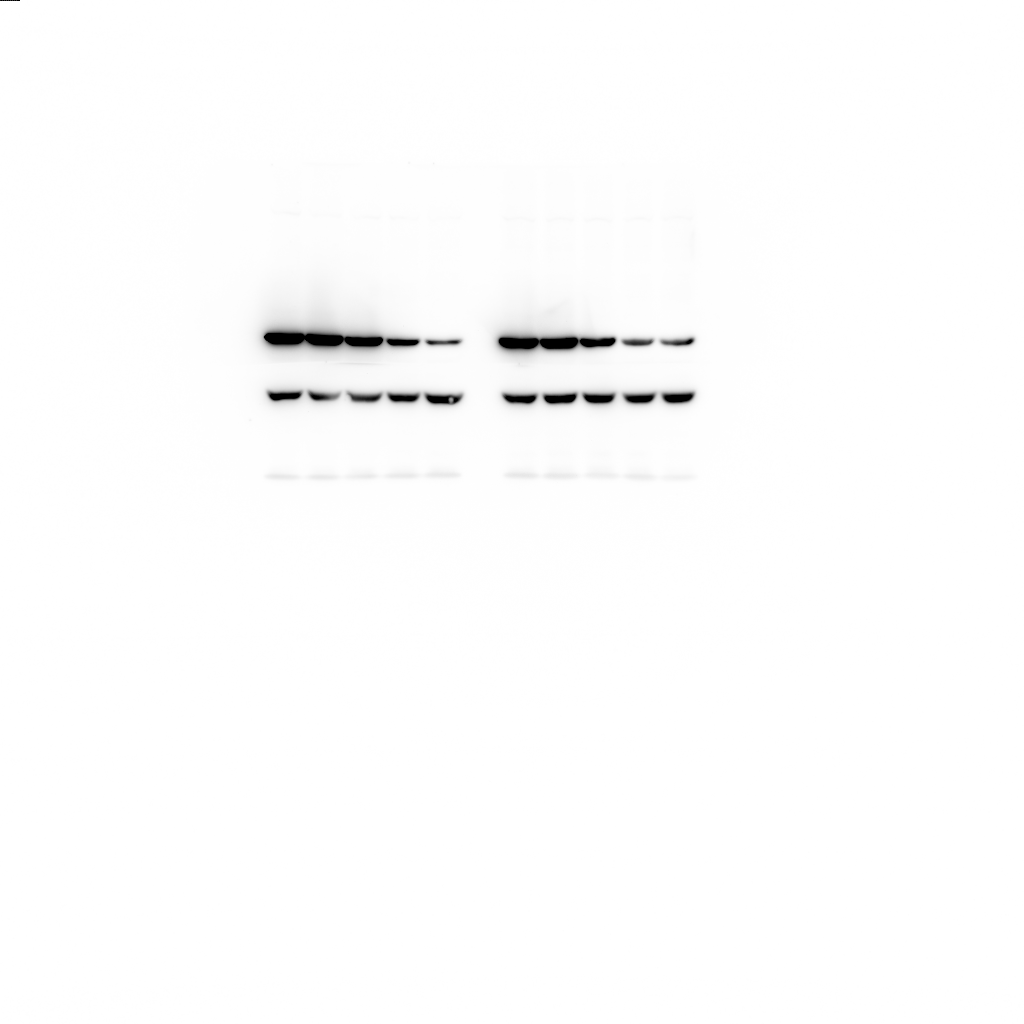

Supplement: Figure 6—source data 2. [file elife-107104-fig6-data2.zip › Figure 6-source data 2. Original files for western blot analysis/Figure 6C-2.tif]

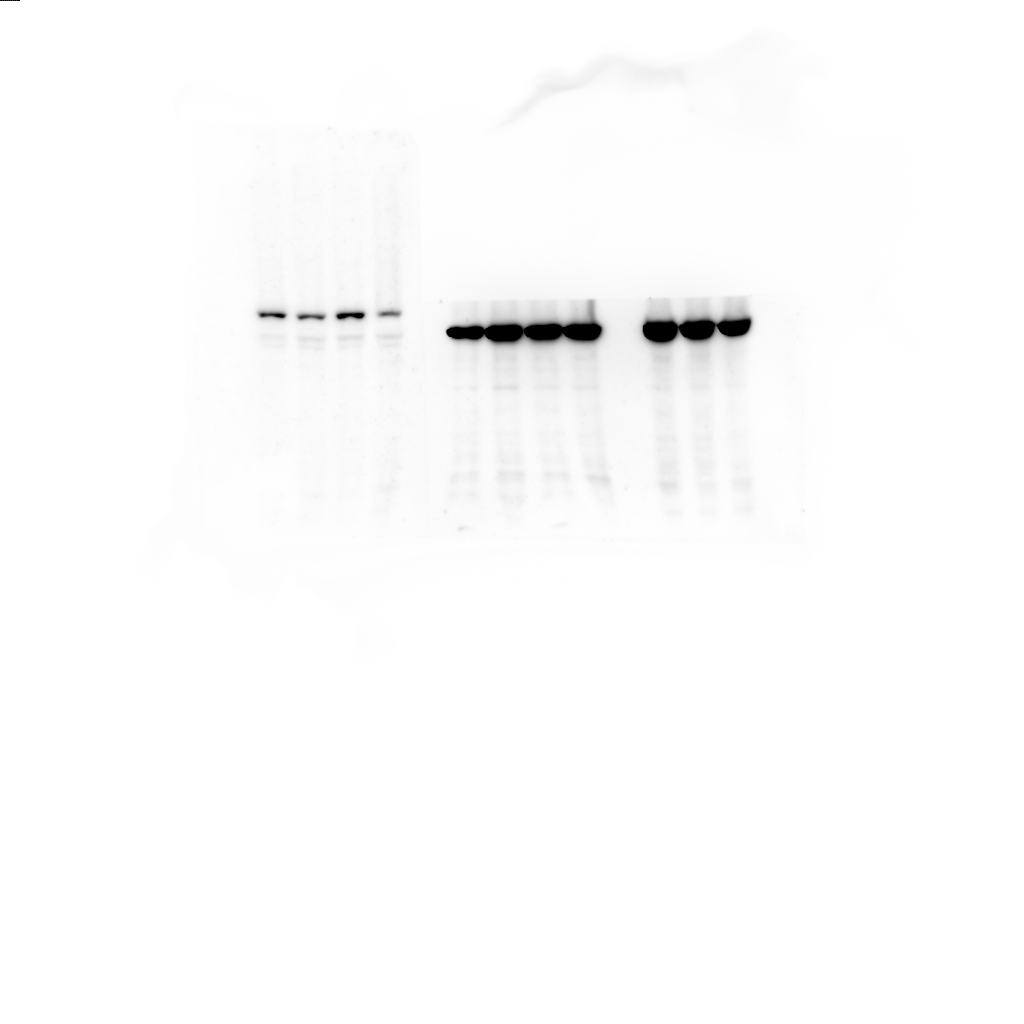

Supplement: Figure 6—source data 2. [file elife-107104-fig6-data2.zip › Figure 6-source data 2. Original files for western blot analysis/Figure 6E-1.TIF]

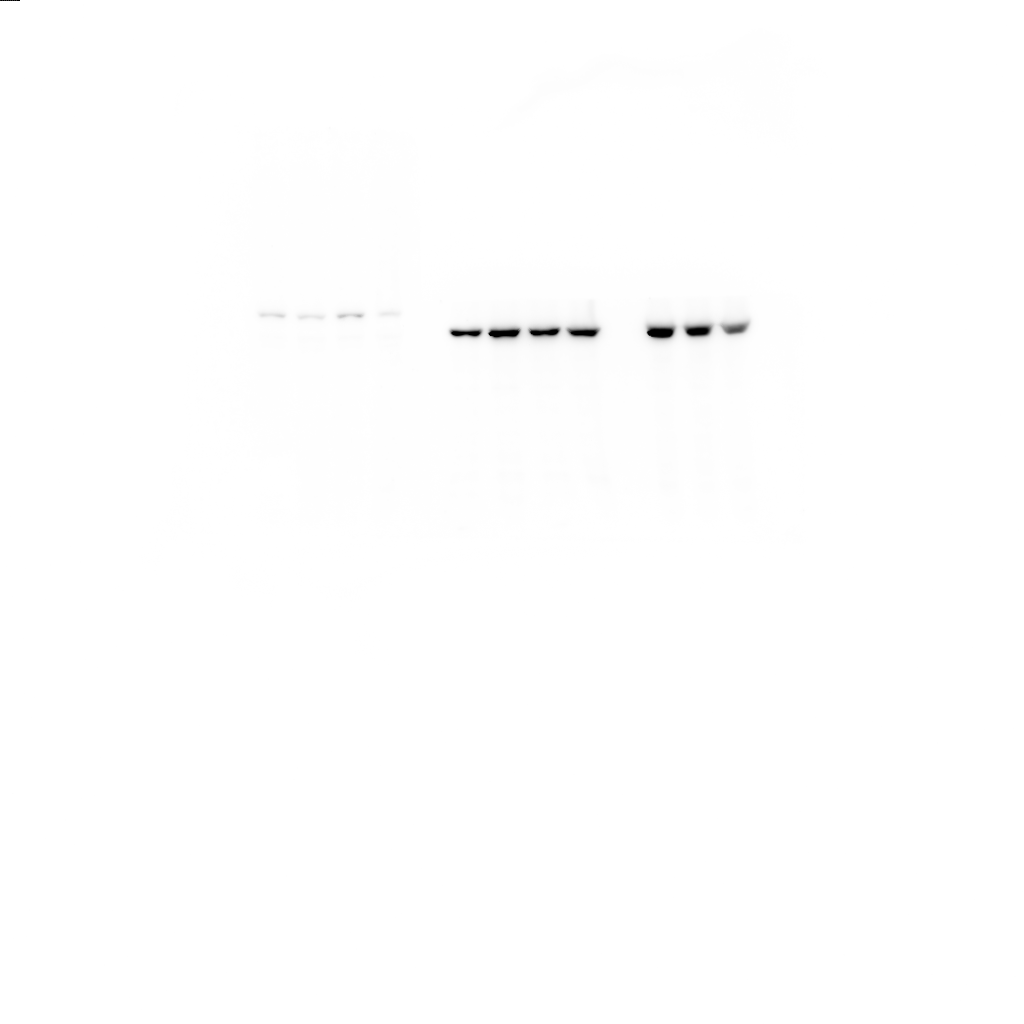

Supplement: Figure 6—source data 2. [file elife-107104-fig6-data2.zip › Figure 6-source data 2. Original files for western blot analysis/Figure 6E-2.TIF]

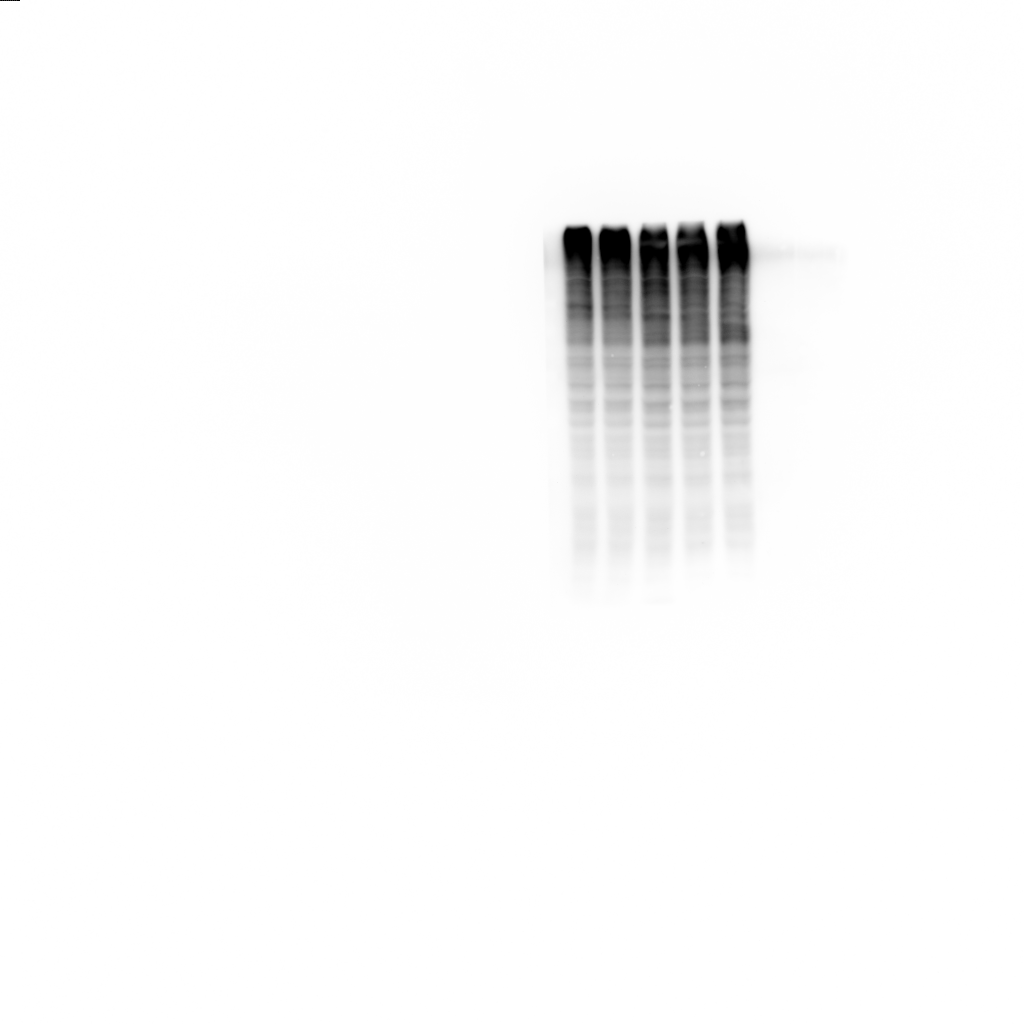

Supplement: Figure 6—source data 2. [file elife-107104-fig6-data2.zip › Figure 6-source data 2. Original files for western blot analysis/Figure 6F-1.TIF]

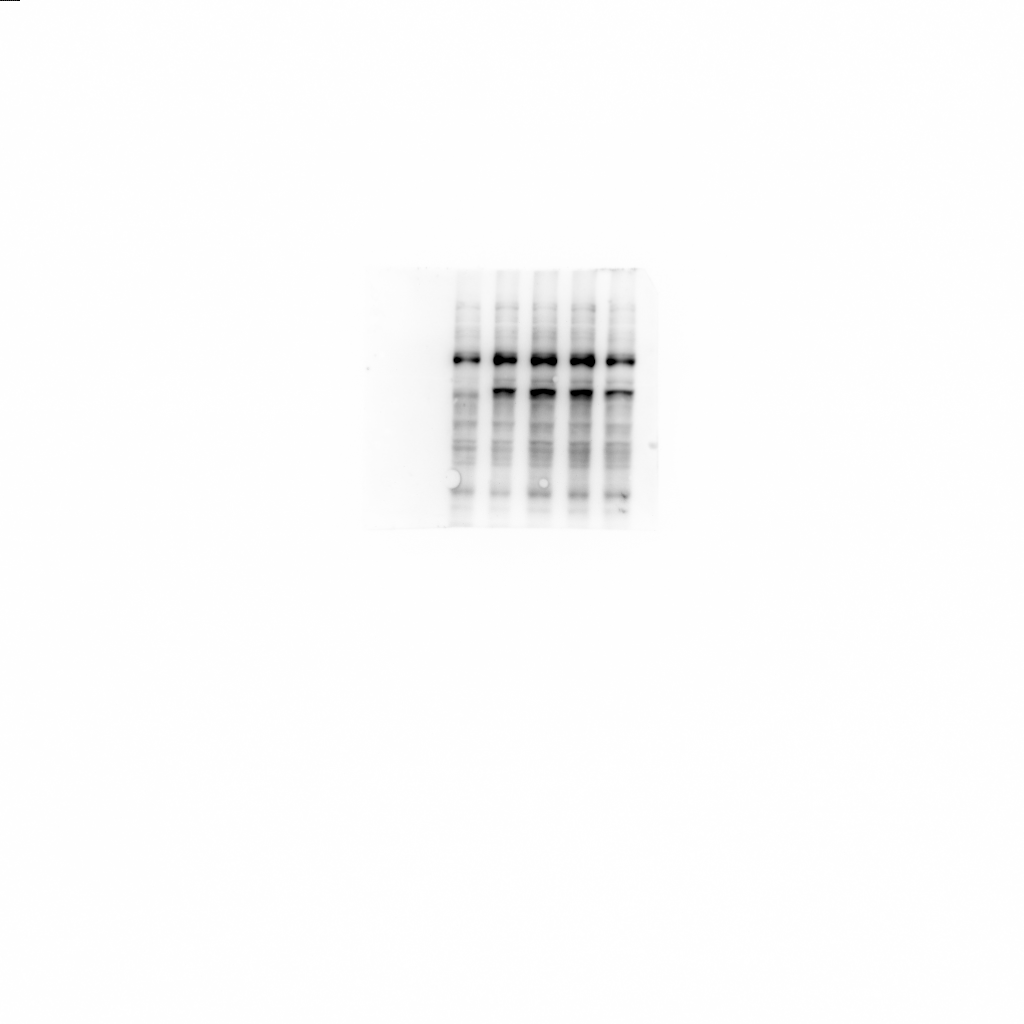

Supplement: Figure 6—source data 2. [file elife-107104-fig6-data2.zip › Figure 6-source data 2. Original files for western blot analysis/Figure 6F-2.tif]

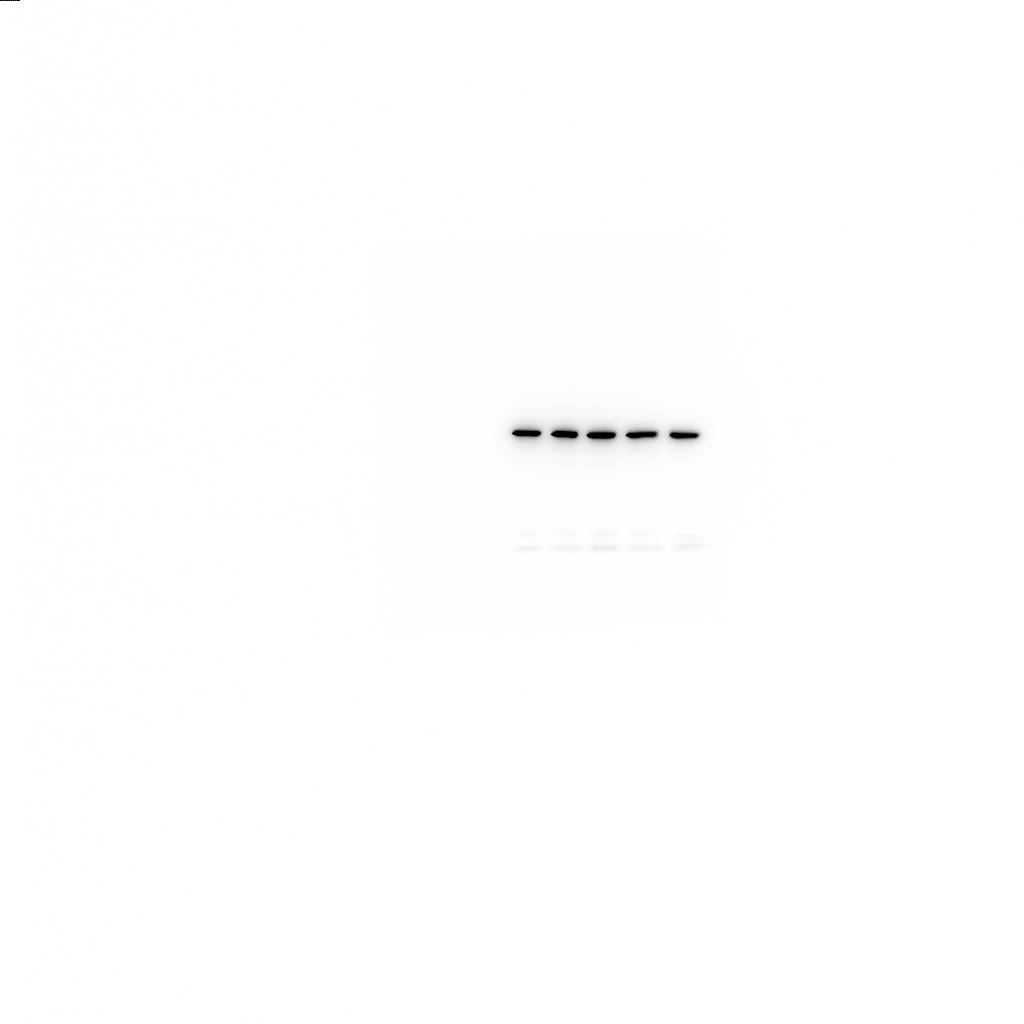

Supplement: Figure 6—source data 2. [file elife-107104-fig6-data2.zip › Figure 6-source data 2. Original files for western blot analysis/Figure 6F-3.TIF]

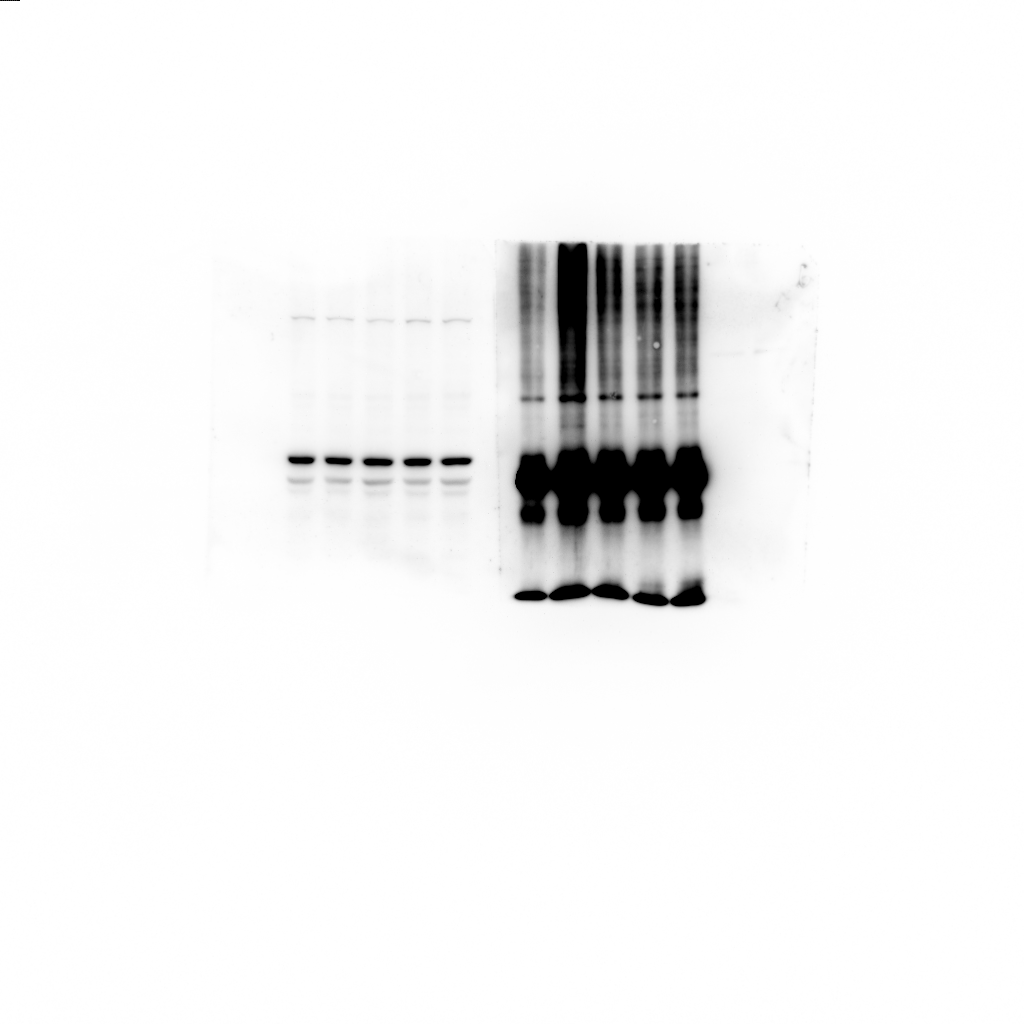

Supplement: Figure 6—source data 2. [file elife-107104-fig6-data2.zip › Figure 6-source data 2. Original files for western blot analysis/Figure 6F-4.tif]

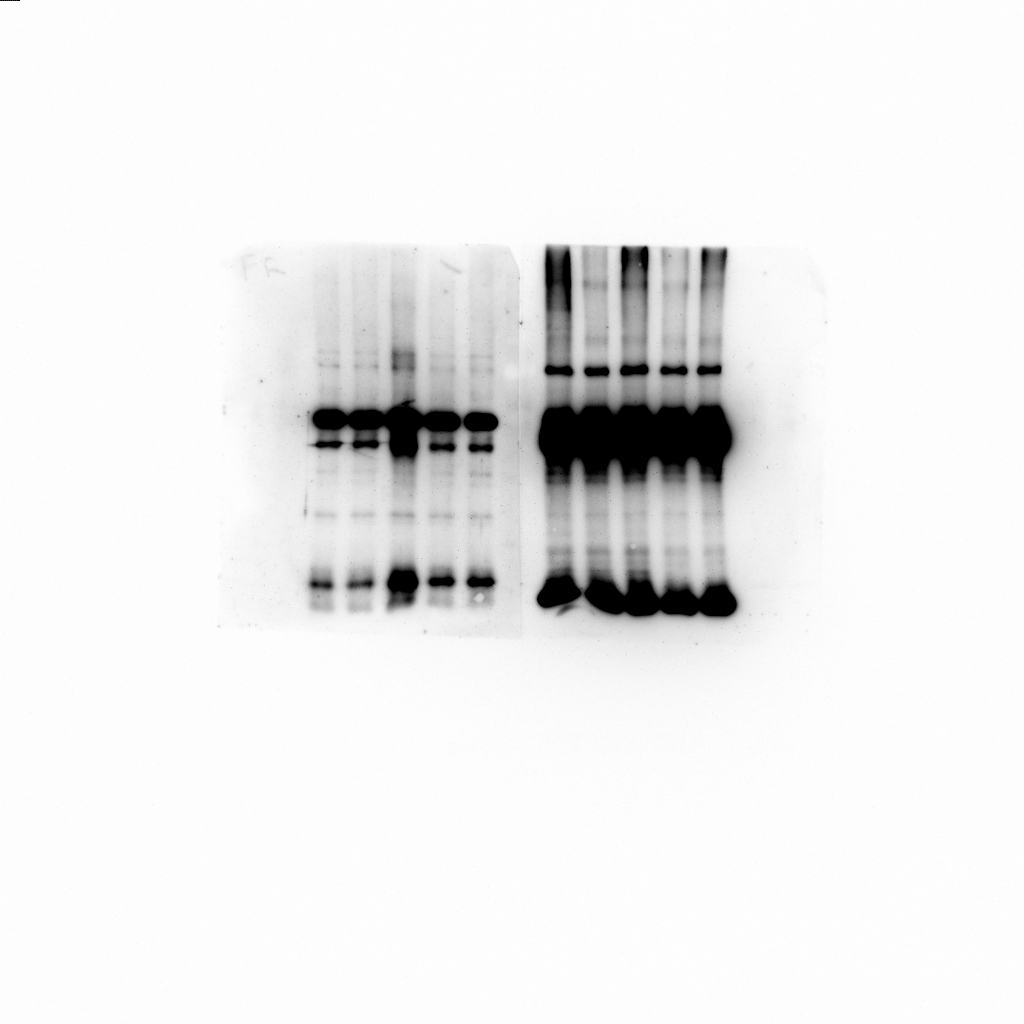

Supplement: Figure 6—source data 2. [file elife-107104-fig6-data2.zip › Figure 6-source data 2. Original files for western blot analysis/Figure 6G-1.tif]

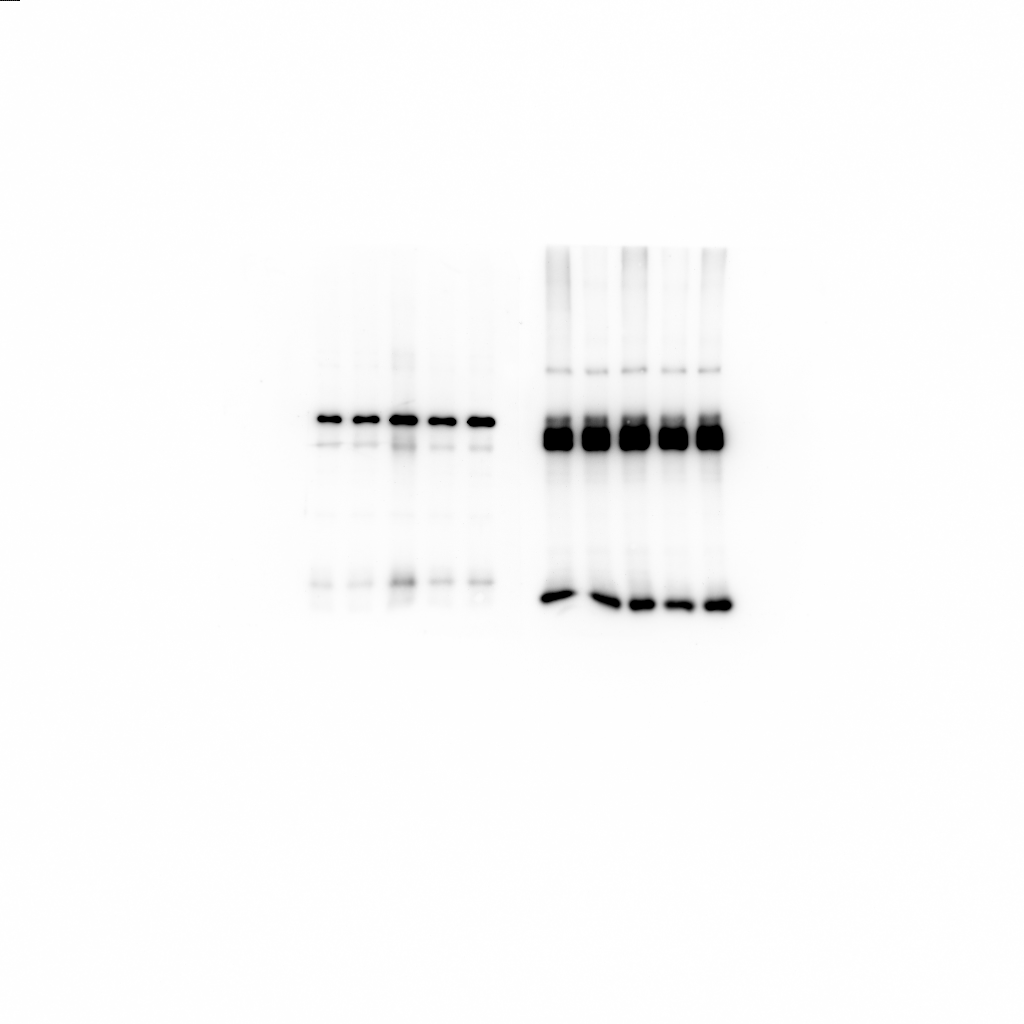

Supplement: Figure 6—source data 2. [file elife-107104-fig6-data2.zip › Figure 6-source data 2. Original files for western blot analysis/Figure 6G-2.tif]

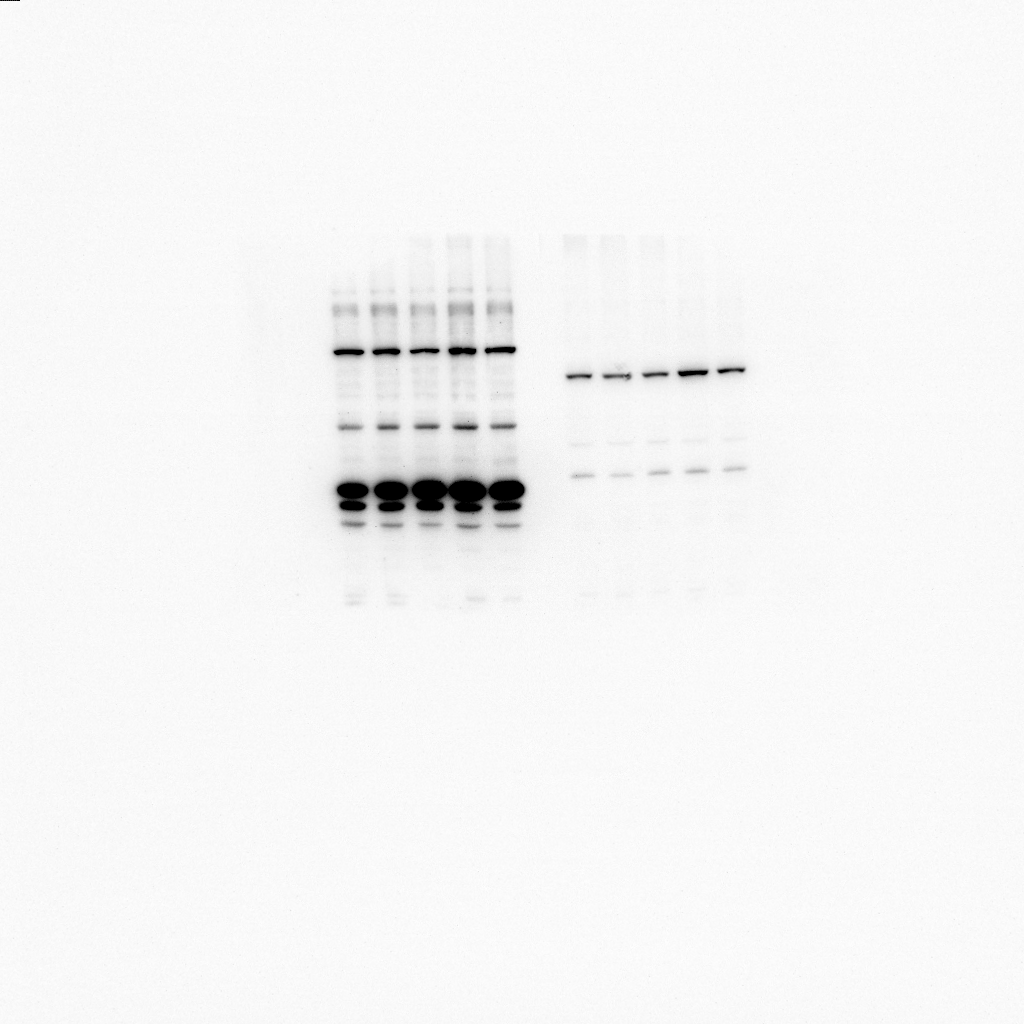

Supplement: Figure 6—source data 2. [file elife-107104-fig6-data2.zip › Figure 6-source data 2. Original files for western blot analysis/Figure 6G-3.TIF]

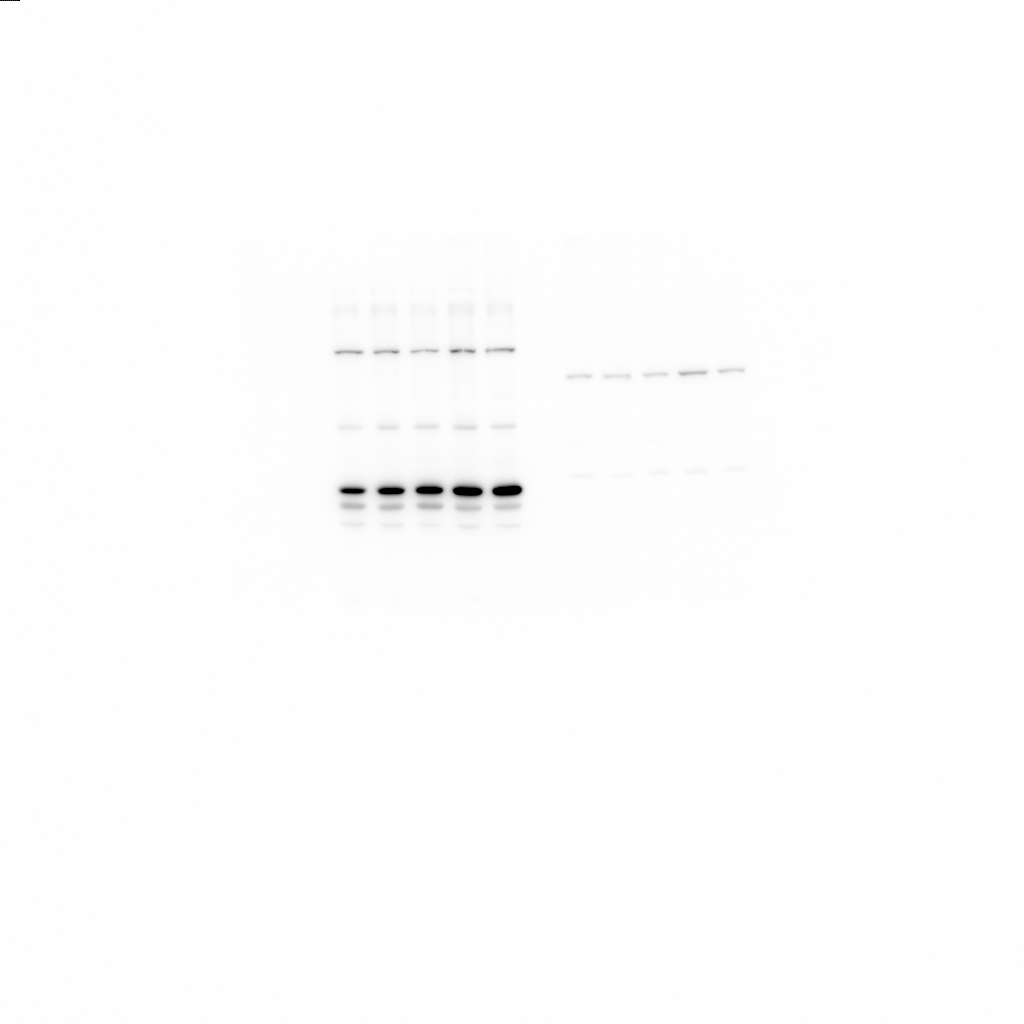

Supplement: Figure 6—source data 2. [file elife-107104-fig6-data2.zip › Figure 6-source data 2. Original files for western blot analysis/Figure 6G-4.TIF]

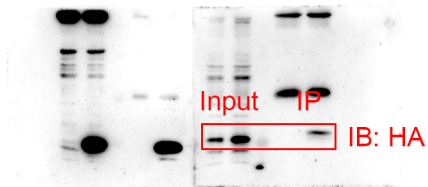

Supplement: Figure 6—figure supplement 1—source data 1. [file elife-107104-fig6-figsupp1-data1.zip › Figure 6-figure supplement 1-source data 1. PDF file containing original western blots, indicating the relevant bands and treatments/Figure 6-figure supplement 1A-1.pdf]

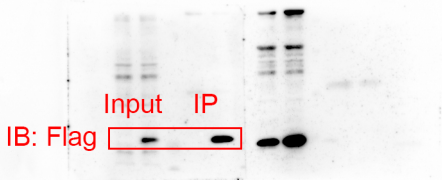

Supplement: Figure 6—figure supplement 1—source data 1. [file elife-107104-fig6-figsupp1-data1.zip › Figure 6-figure supplement 1-source data 1. PDF file containing original western blots, indicating the relevant bands and treatments/Figure 6-figure supplement 1A-2.pdf]

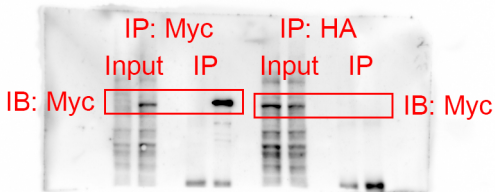

Supplement: Figure 6—figure supplement 1—source data 1. [file elife-107104-fig6-figsupp1-data1.zip › Figure 6-figure supplement 1-source data 1. PDF file containing original western blots, indicating the relevant bands and treatments/Figure 6-figure supplement 1B-1.pdf]

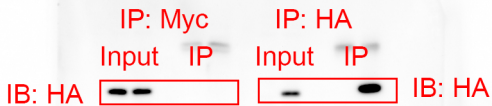

Supplement: Figure 6—figure supplement 1—source data 1. [file elife-107104-fig6-figsupp1-data1.zip › Figure 6-figure supplement 1-source data 1. PDF file containing original western blots, indicating the relevant bands and treatments/Figure 6-figure supplement 1B-2.pdf]

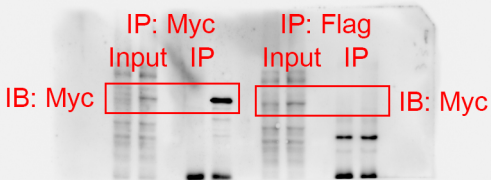

Supplement: Figure 6—figure supplement 1—source data 1. [file elife-107104-fig6-figsupp1-data1.zip › Figure 6-figure supplement 1-source data 1. PDF file containing original western blots, indicating the relevant bands and treatments/Figure 6-figure supplement 1C-1.pdf]

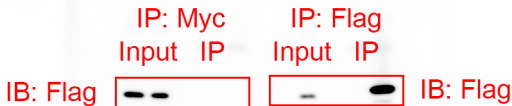

Supplement: Figure 6—figure supplement 1—source data 1. [file elife-107104-fig6-figsupp1-data1.zip › Figure 6-figure supplement 1-source data 1. PDF file containing original western blots, indicating the relevant bands and treatments/Figure 6-figure supplement 1C-2.pdf]

IP: HA                      IP: Myc  
Input    IP    Input    IP  
IB: Myc                                           IB: Myc

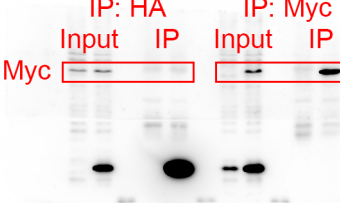

Supplement: Figure 6—figure supplement 1—source data 1. [file elife-107104-fig6-figsupp1-data1.zip › Figure 6-figure supplement 1-source data 1. PDF file containing original western blots, indicating the relevant bands and treatments/Figure 6-figure supplement 1D-1.pdf]

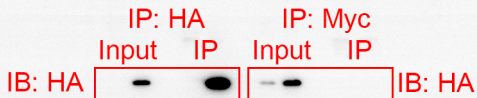

Supplement: Figure 6—figure supplement 1—source data 1. [file elife-107104-fig6-figsupp1-data1.zip › Figure 6-figure supplement 1-source data 1. PDF file containing original western blots, indicating the relevant bands and treatments/Figure 6-figure supplement 1D-2.pdf]

IP: HA      IP: Myc  
Input   IP   Input   IP

IB: Myc 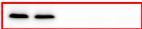 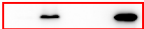 IB: Myc

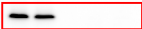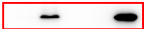

Supplement: Figure 6—figure supplement 1—source data 1. [file elife-107104-fig6-figsupp1-data1.zip › Figure 6-figure supplement 1-source data 1. PDF file containing original western blots, indicating the relevant bands and treatments/Figure 6-figure supplement 1E-1.pdf]

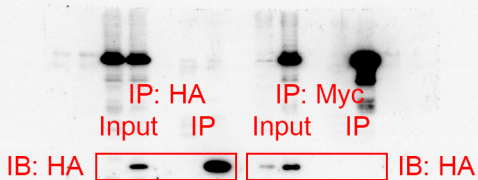

Supplement: Figure 6—figure supplement 1—source data 1. [file elife-107104-fig6-figsupp1-data1.zip › Figure 6-figure supplement 1-source data 1. PDF file containing original western blots, indicating the relevant bands and treatments/Figure 6-figure supplement 1E-2.pdf]

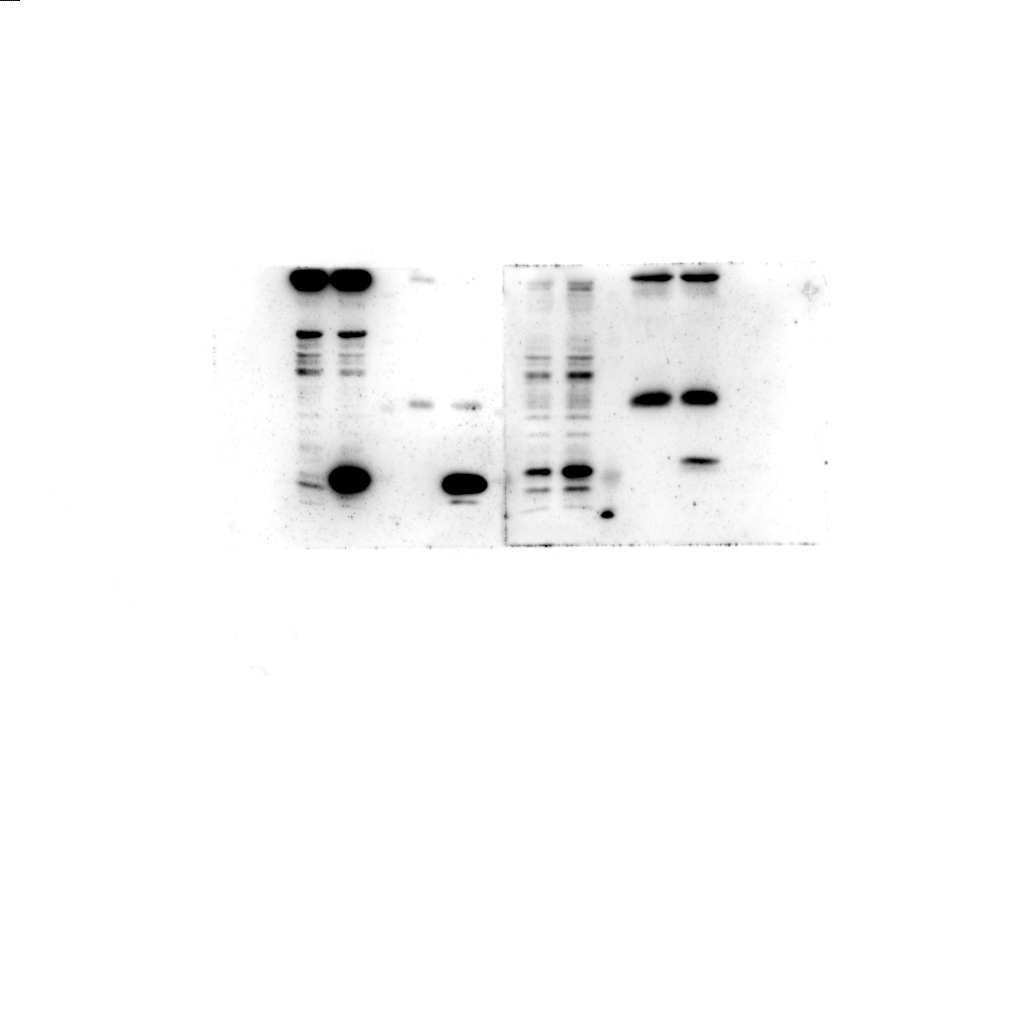

Supplement: Figure 6—figure supplement 1—source data 2. [file elife-107104-fig6-figsupp1-data2.zip › Figure 6-figure supplement 1-source data 2. Original files for western blot analysis/Figure 6-figure supplement 1A-1.tif]

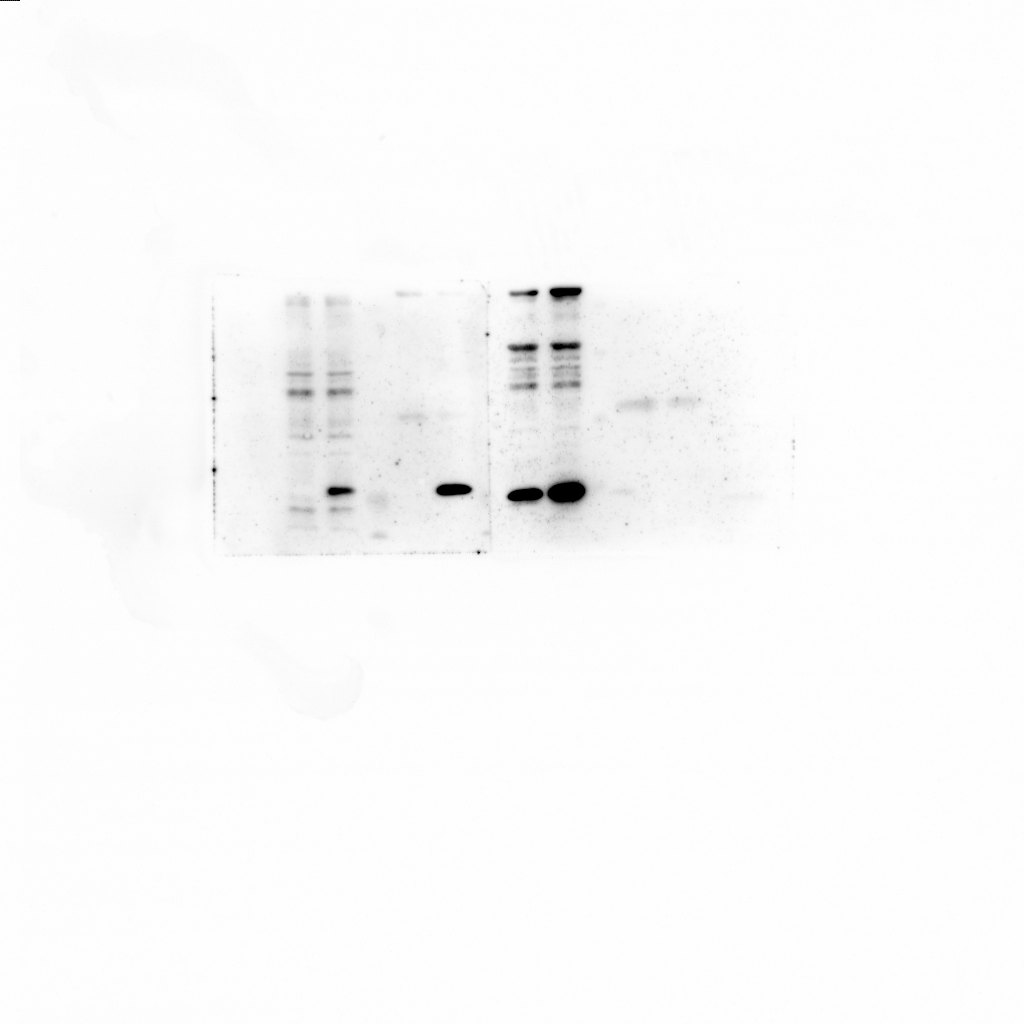

Supplement: Figure 6—figure supplement 1—source data 2. [file elife-107104-fig6-figsupp1-data2.zip › Figure 6-figure supplement 1-source data 2. Original files for western blot analysis/Figure 6-figure supplement 1A-2.tif]

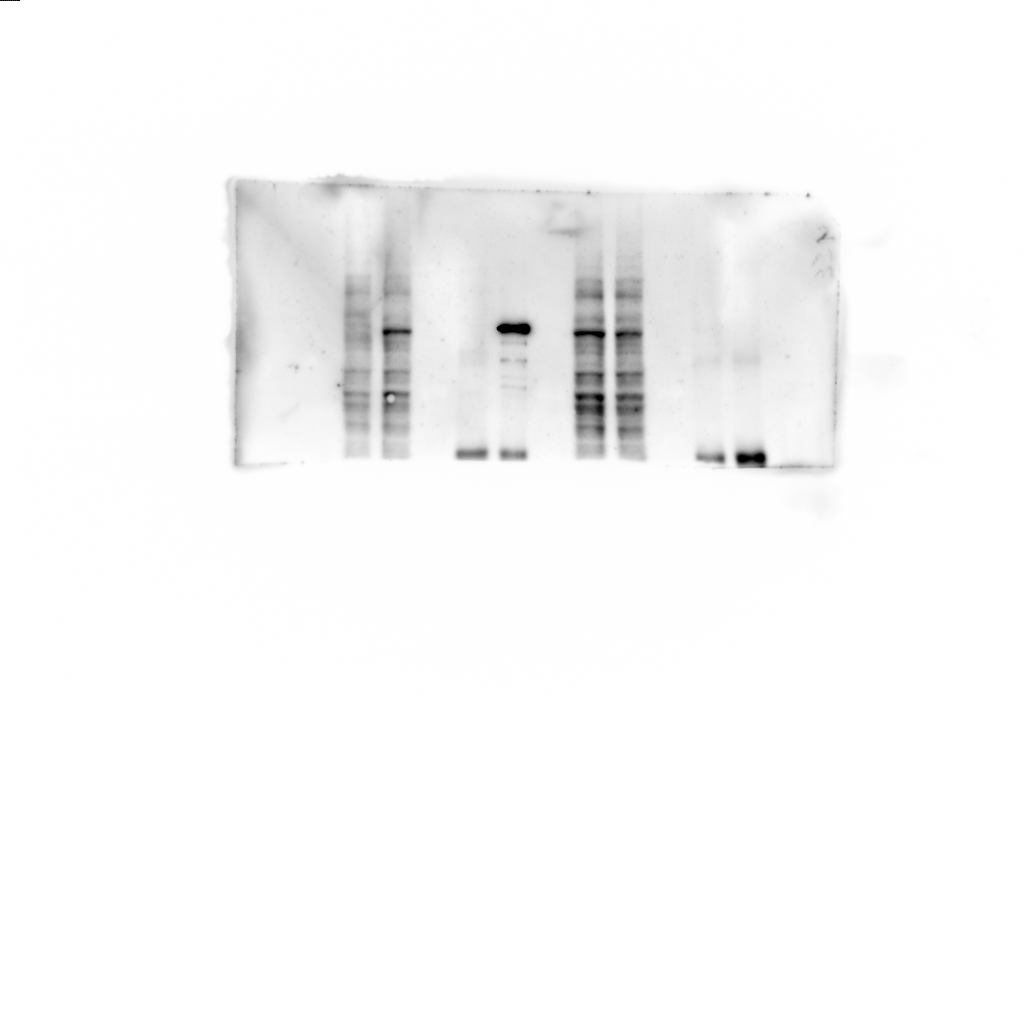

Supplement: Figure 6—figure supplement 1—source data 2. [file elife-107104-fig6-figsupp1-data2.zip › Figure 6-figure supplement 1-source data 2. Original files for western blot analysis/Figure 6-figure supplement 1B-1.tif]

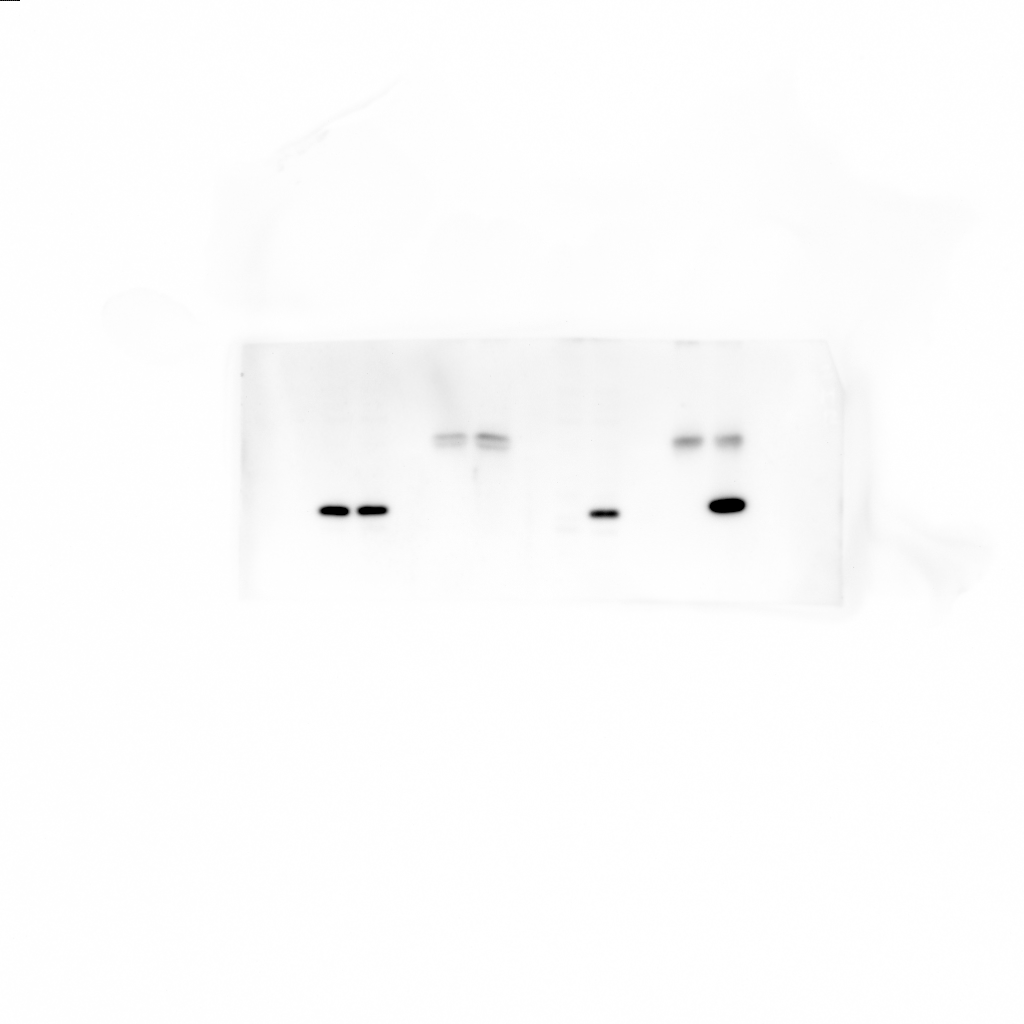

Supplement: Figure 6—figure supplement 1—source data 2. [file elife-107104-fig6-figsupp1-data2.zip › Figure 6-figure supplement 1-source data 2. Original files for western blot analysis/Figure 6-figure supplement 1B-2.tif]

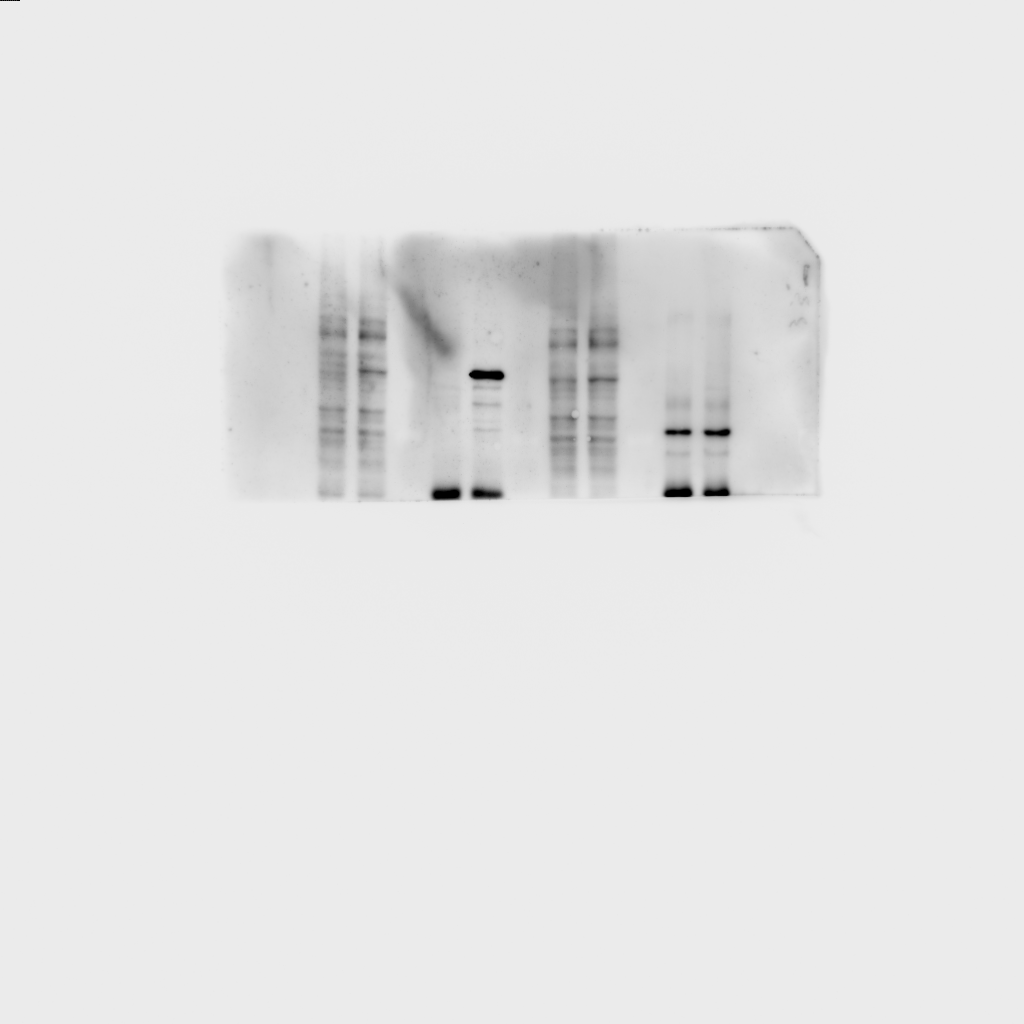

Supplement: Figure 6—figure supplement 1—source data 2. [file elife-107104-fig6-figsupp1-data2.zip › Figure 6-figure supplement 1-source data 2. Original files for western blot analysis/Figure 6-figure supplement 1C-1.TIF]

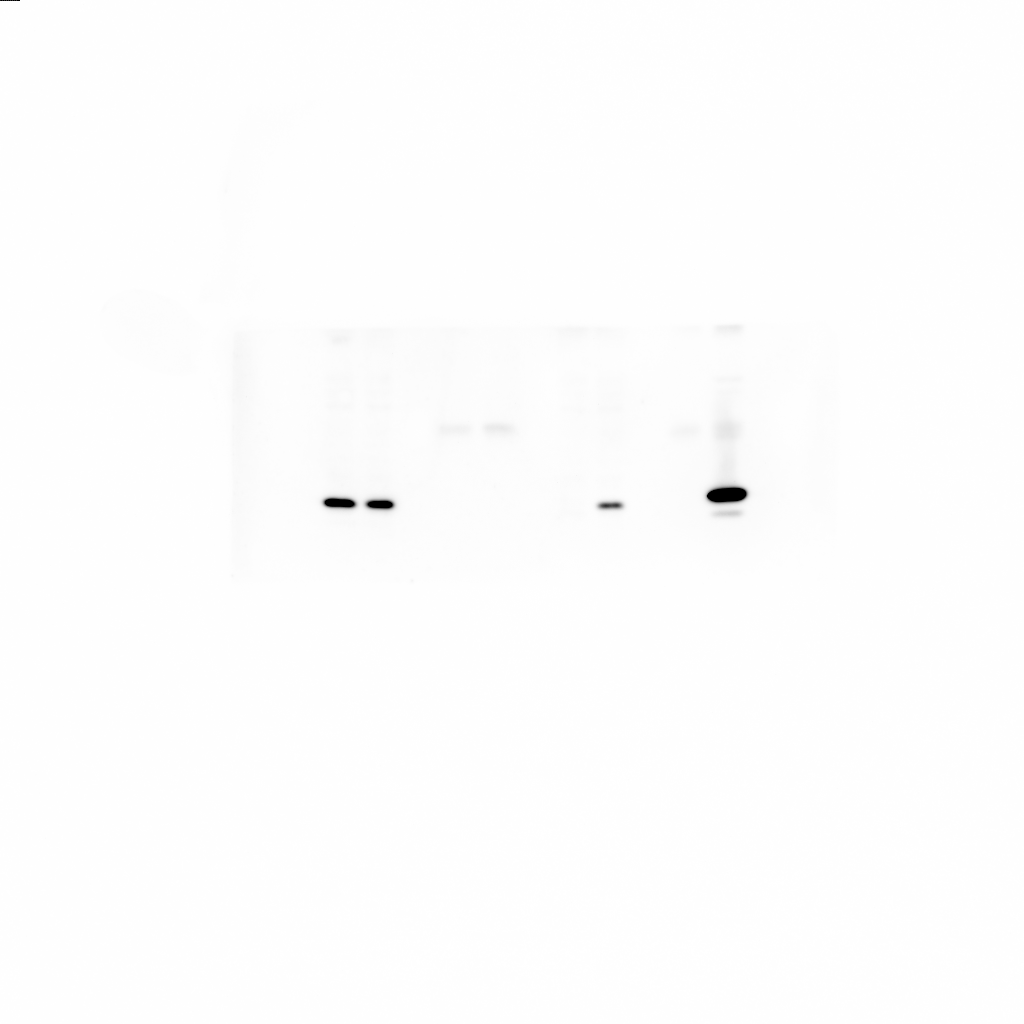

Supplement: Figure 6—figure supplement 1—source data 2. [file elife-107104-fig6-figsupp1-data2.zip › Figure 6-figure supplement 1-source data 2. Original files for western blot analysis/Figure 6-figure supplement 1C-2.TIF]

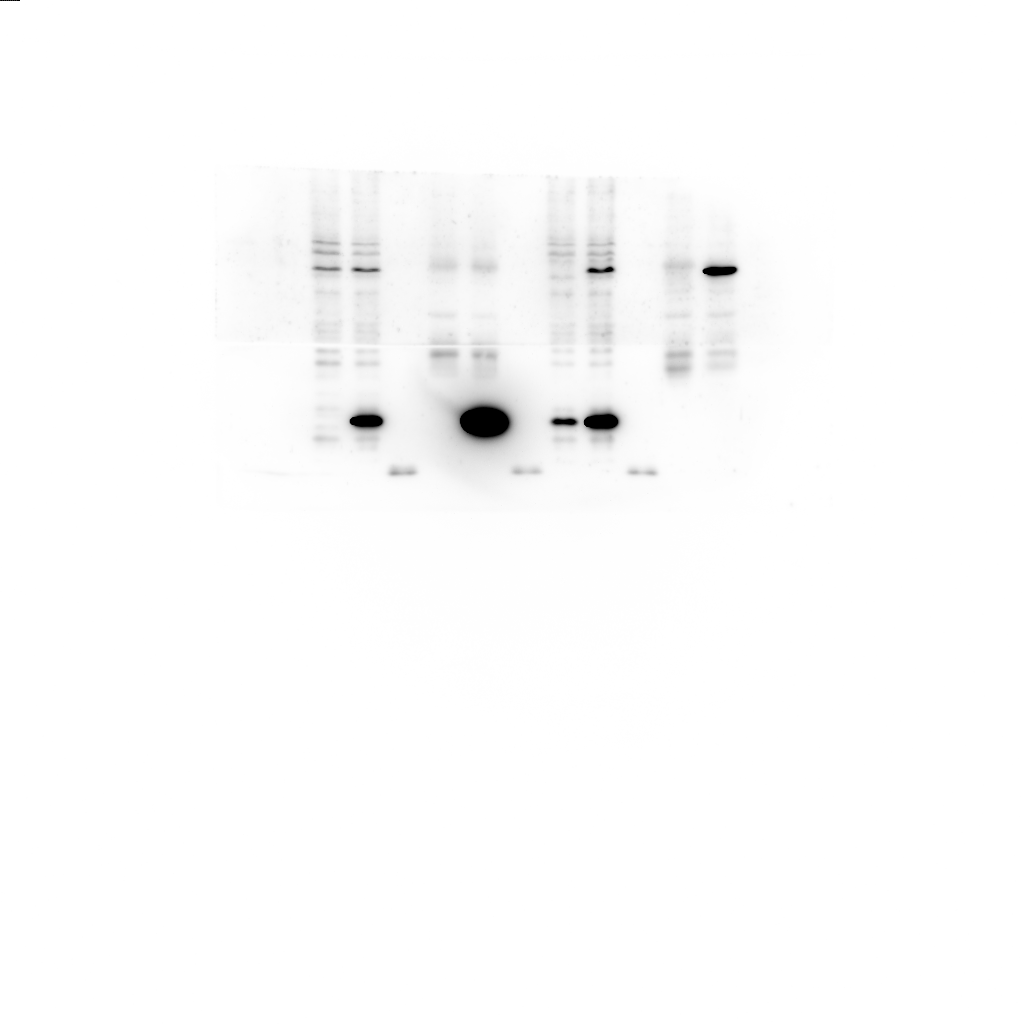

Supplement: Figure 6—figure supplement 1—source data 2. [file elife-107104-fig6-figsupp1-data2.zip › Figure 6-figure supplement 1-source data 2. Original files for western blot analysis/Figure 6-figure supplement 1D-1.tif]

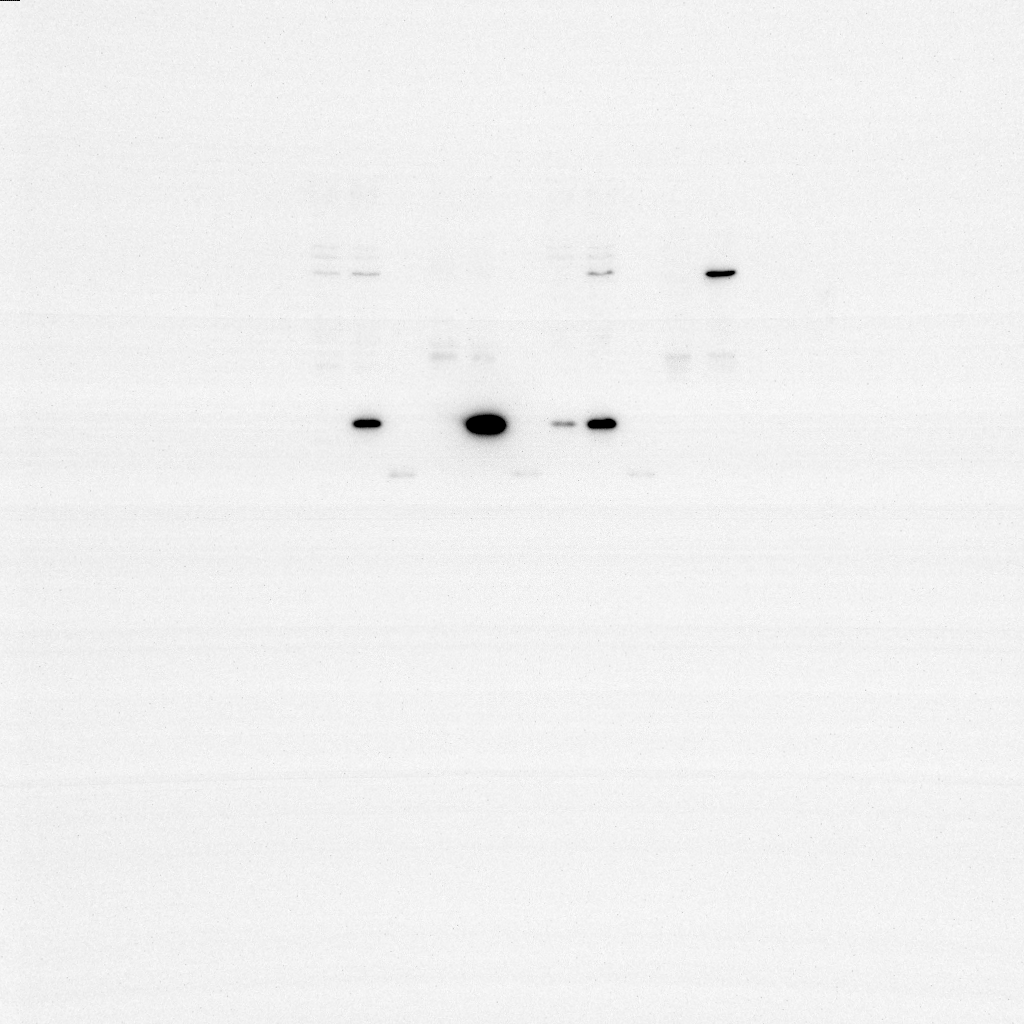

Supplement: Figure 6—figure supplement 1—source data 2. [file elife-107104-fig6-figsupp1-data2.zip › Figure 6-figure supplement 1-source data 2. Original files for western blot analysis/Figure 6-figure supplement 1D-2.tif]

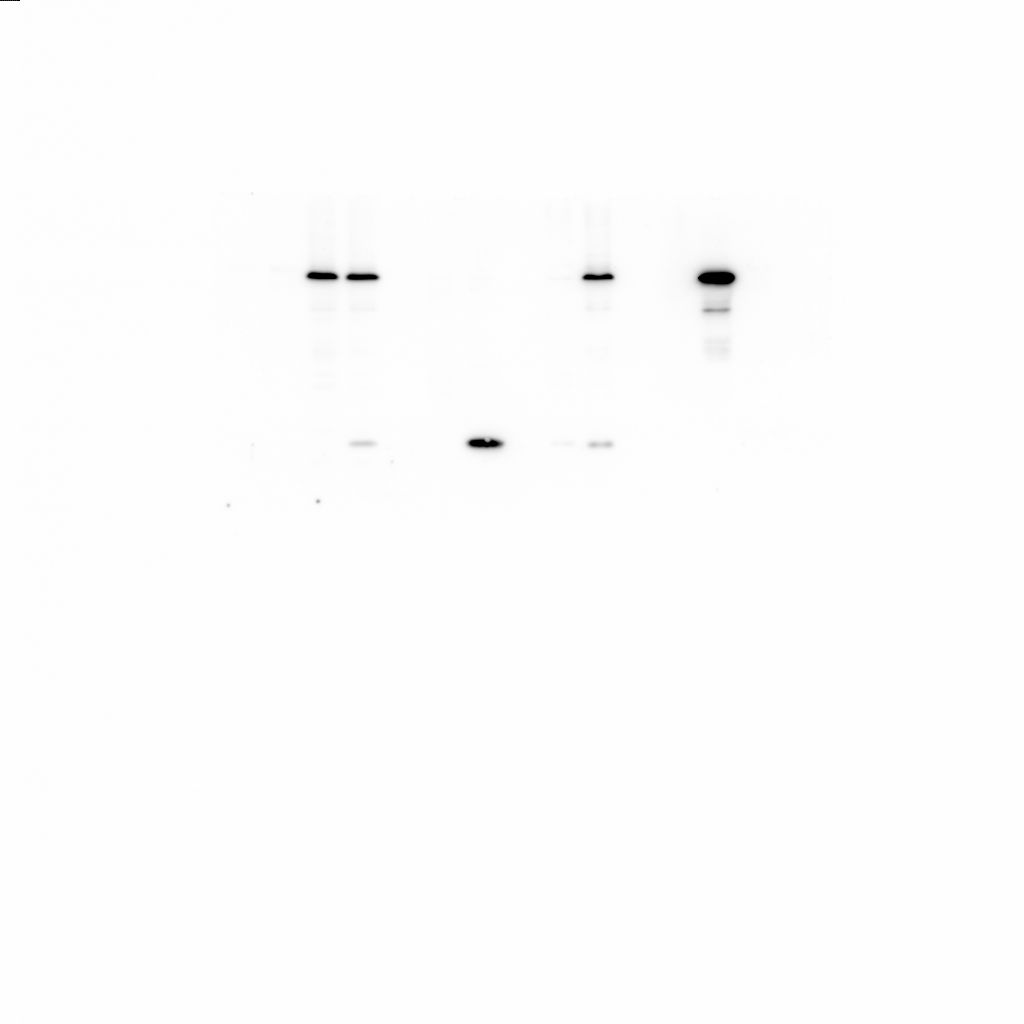

Supplement: Figure 6—figure supplement 1—source data 2. [file elife-107104-fig6-figsupp1-data2.zip › Figure 6-figure supplement 1-source data 2. Original files for western blot analysis/Figure 6-figure supplement 1E-1.TIF]

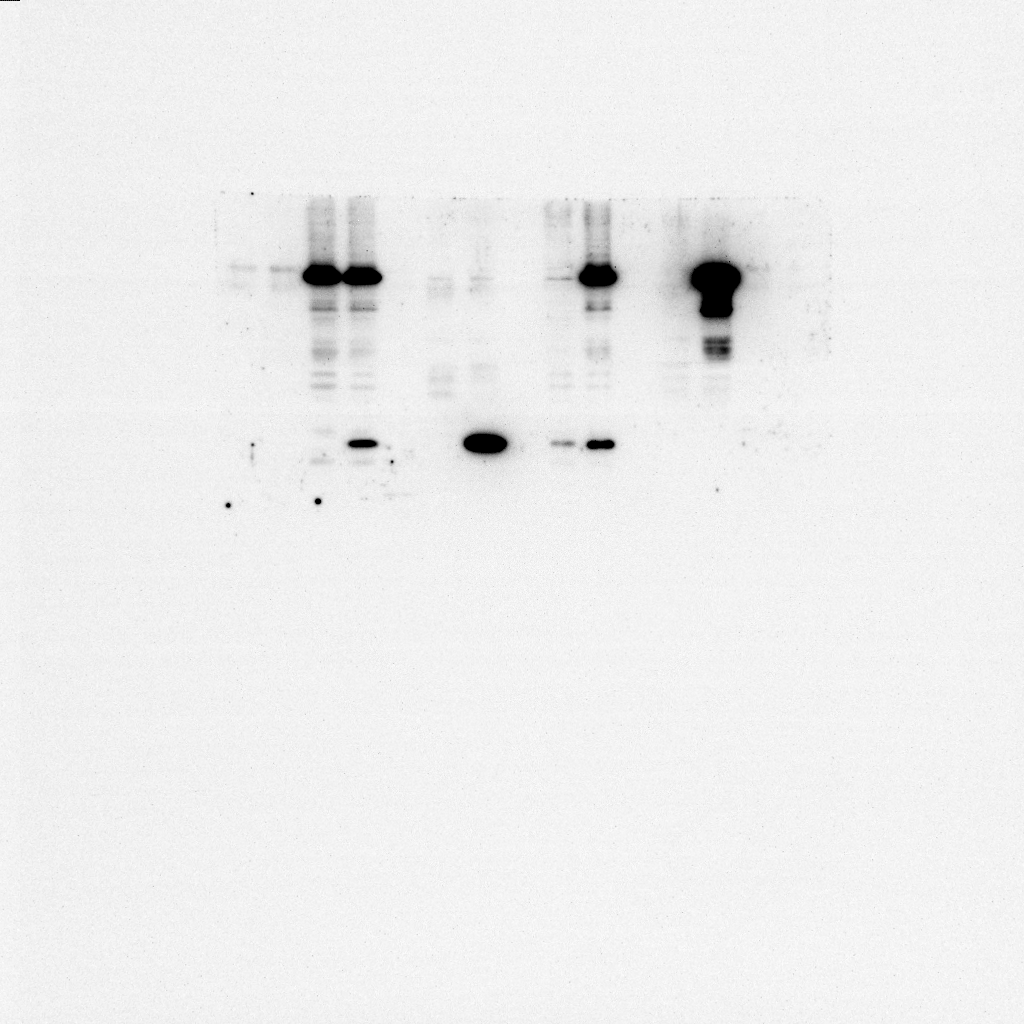

Supplement: Figure 6—figure supplement 1—source data 2. [file elife-107104-fig6-figsupp1-data2.zip › Figure 6-figure supplement 1-source data 2. Original files for western blot analysis/Figure 6-figure supplement 1E-2.TIF]

Flag-Ben HA-Uev1A

IB:  $\beta$ -Actin 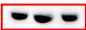 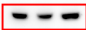 IB:  $\beta$ -Actin

IB: Flag 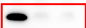 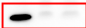 IB: HA

Supplement: Figure 6—figure supplement 2—source data 1. [file elife-107104-fig6-figsupp2-data1.zip › Figure 6-figure supplement 2-source data 1. PDF file containing original western blots, indicating the relevant bands and treatments/Figure 6-figure supplement 2A, B.pdf]

Myc-Cdc27

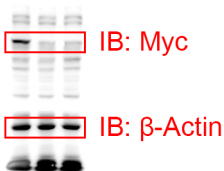

Supplement: Figure 6—figure supplement 2—source data 1. [file elife-107104-fig6-figsupp2-data1.zip › Figure 6-figure supplement 2-source data 1. PDF file containing original western blots, indicating the relevant bands and treatments/Figure 6-figure supplement 2C.pdf]

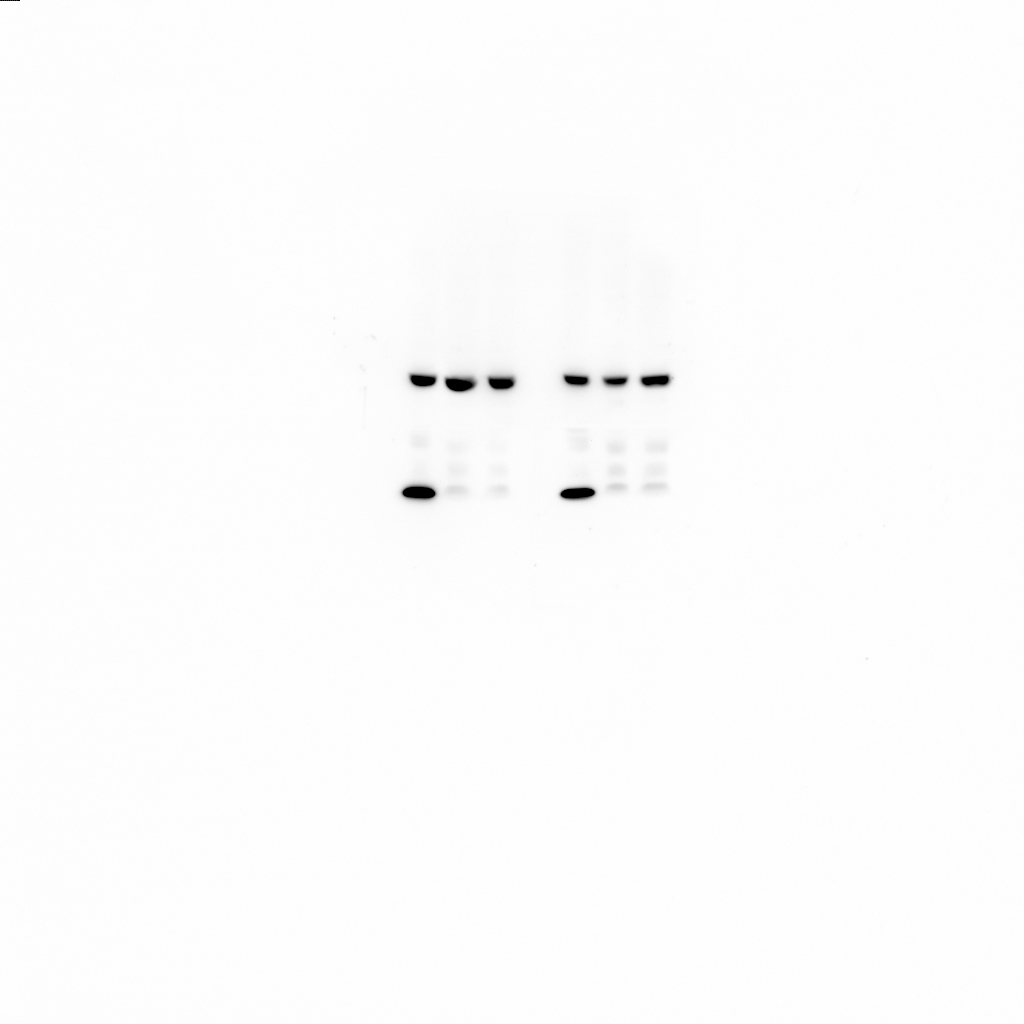

Supplement: Figure 6—figure supplement 2—source data 2. [file elife-107104-fig6-figsupp2-data2.zip › Figure 6-figure supplement 2-source data 2. Original files for western blot analysis/Figure 6-figure supplement 2A, B.TIF]

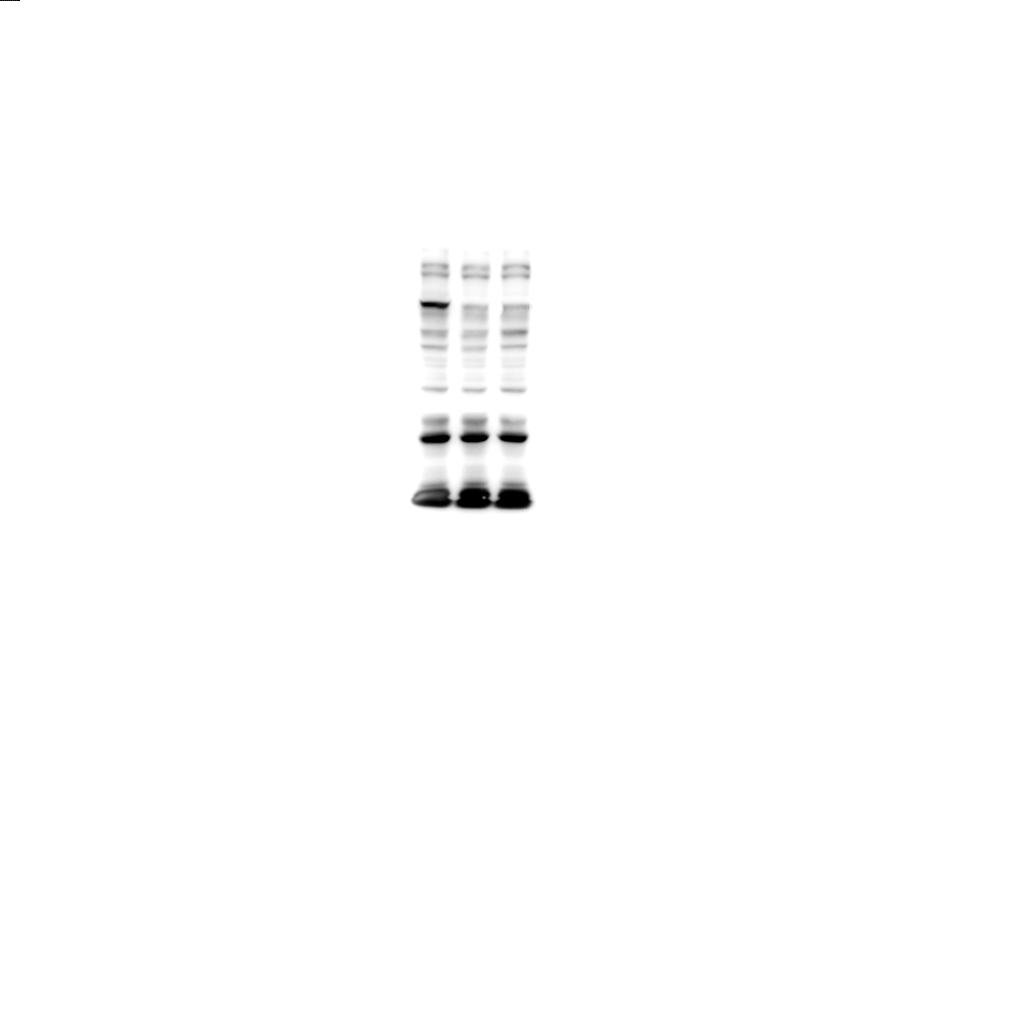

Supplement: Figure 6—figure supplement 2—source data 2. [file elife-107104-fig6-figsupp2-data2.zip › Figure 6-figure supplement 2-source data 2. Original files for western blot analysis/Figure 6-figure supplement 2C.tif]

SW480 HCT116

IB:  $\beta$ -Actin 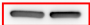 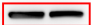

IB: UBE2V1 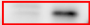 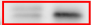

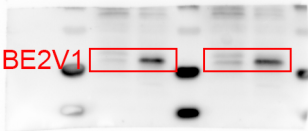

Supplement: Figure 9—figure supplement 1—source data 1. [file elife-107104-fig9-figsupp1-data1.zip › Figure 9-figure supplement 1-source data 1. PDF file containing original western blots, indicating the relevant bands and treatments/Figure 9-figure supplement 1A-1.pdf]

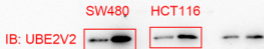

Supplement: Figure 9—figure supplement 1—source data 1. [file elife-107104-fig9-figsupp1-data1.zip › Figure 9-figure supplement 1-source data 1. PDF file containing original western blots, indicating the relevant bands and treatments/Figure 9-figure supplement 1A-2.pdf]

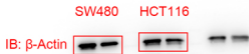

Supplement: Figure 9—figure supplement 1—source data 1. [file elife-107104-fig9-figsupp1-data1.zip › Figure 9-figure supplement 1-source data 1. PDF file containing original western blots, indicating the relevant bands and treatments/Figure 9-figure supplement 1A-3.pdf]

UBE2V1-OE

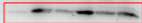

IB: UBE2V1

Supplement: Figure 9—figure supplement 1—source data 1. [file elife-107104-fig9-figsupp1-data1.zip › Figure 9-figure supplement 1-source data 1. PDF file containing original western blots, indicating the relevant bands and treatments/Figure 9-figure supplement 1B-1.pdf]

UBE2V1-OE

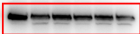

IB:  $\alpha$ -Tubulin

Supplement: Figure 9—figure supplement 1—source data 1. [file elife-107104-fig9-figsupp1-data1.zip › Figure 9-figure supplement 1-source data 1. PDF file containing original western blots, indicating the relevant bands and treatments/Figure 9-figure supplement 1B-2.pdf]

UBE2V2-OE

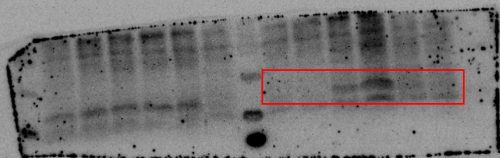

IB: UBE2V2

Supplement: Figure 9—figure supplement 1—source data 1. [file elife-107104-fig9-figsupp1-data1.zip › Figure 9-figure supplement 1-source data 1. PDF file containing original western blots, indicating the relevant bands and treatments/Figure 9-figure supplement 1B-3.pdf]

UBE2V2-OE

IB:  $\alpha$ -Tubulin

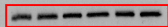

Supplement: Figure 9—figure supplement 1—source data 1. [file elife-107104-fig9-figsupp1-data1.zip › Figure 9-figure supplement 1-source data 1. PDF file containing original western blots, indicating the relevant bands and treatments/Figure 9-figure supplement 1B-4.pdf]

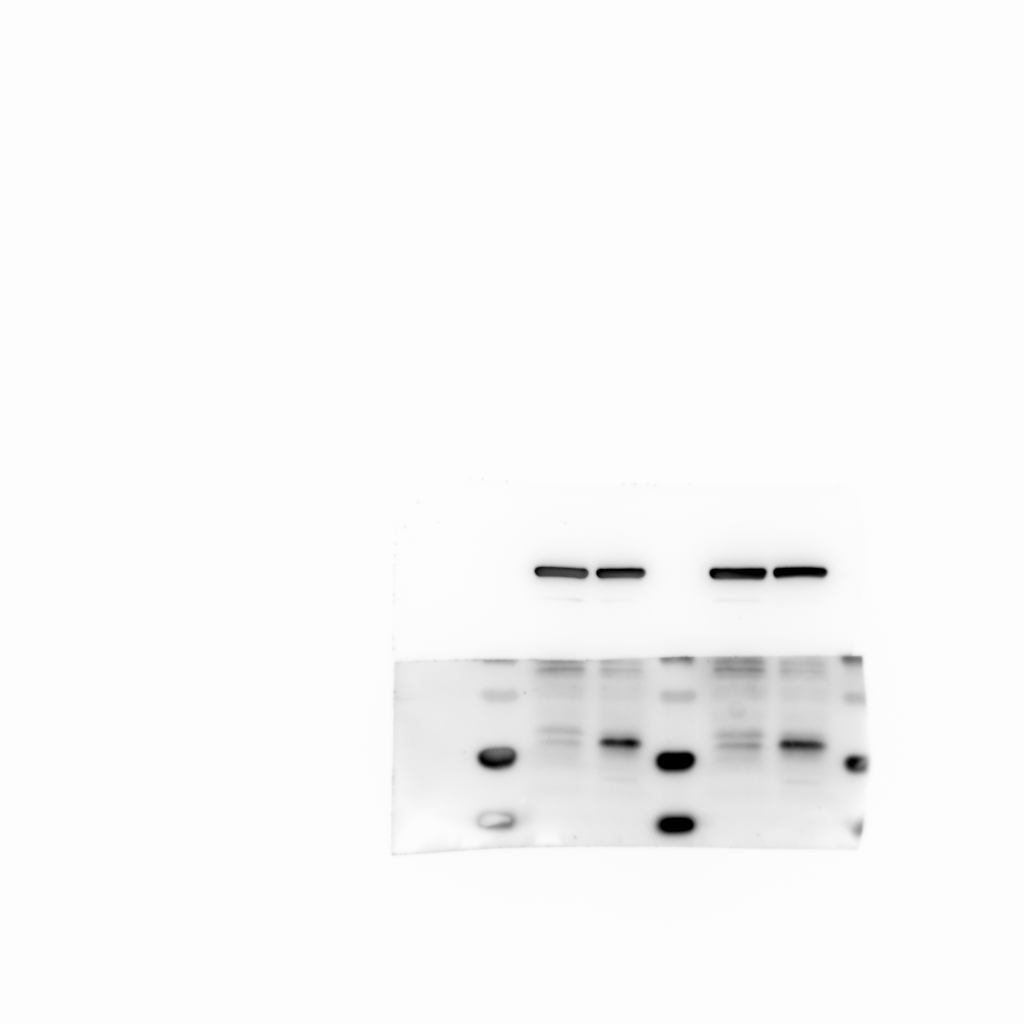

Supplement: Figure 9—figure supplement 1—source data 2. [file elife-107104-fig9-figsupp1-data2.zip › Figure 9-figure supplement 1-source data 2. Original files for western blot analysis/Figure 9-figure supplement 1A-1.tif]

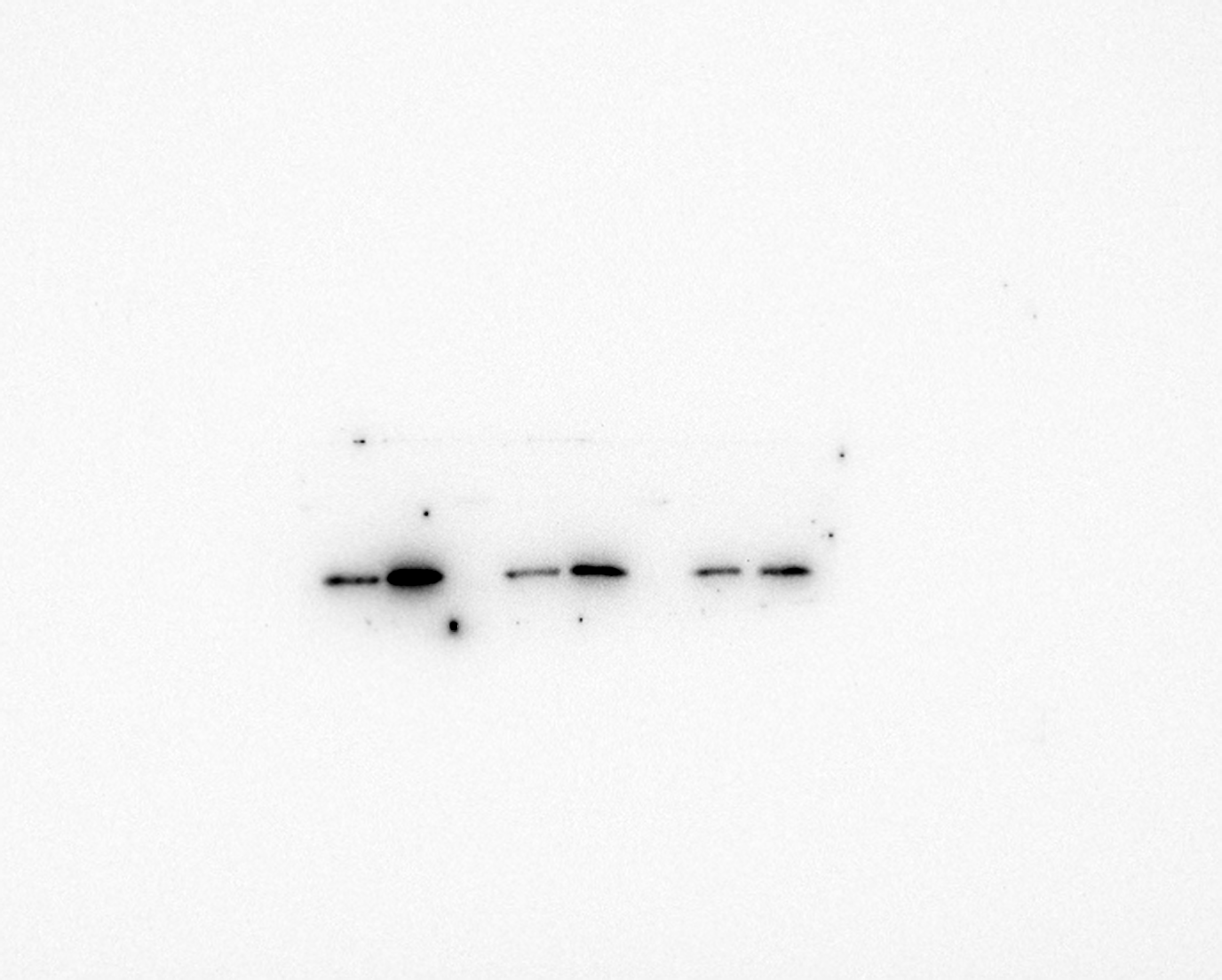

Supplement: Figure 9—figure supplement 1—source data 2. [file elife-107104-fig9-figsupp1-data2.zip › Figure 9-figure supplement 1-source data 2. Original files for western blot analysis/Figure 9-figure supplement 1A-2.tif]

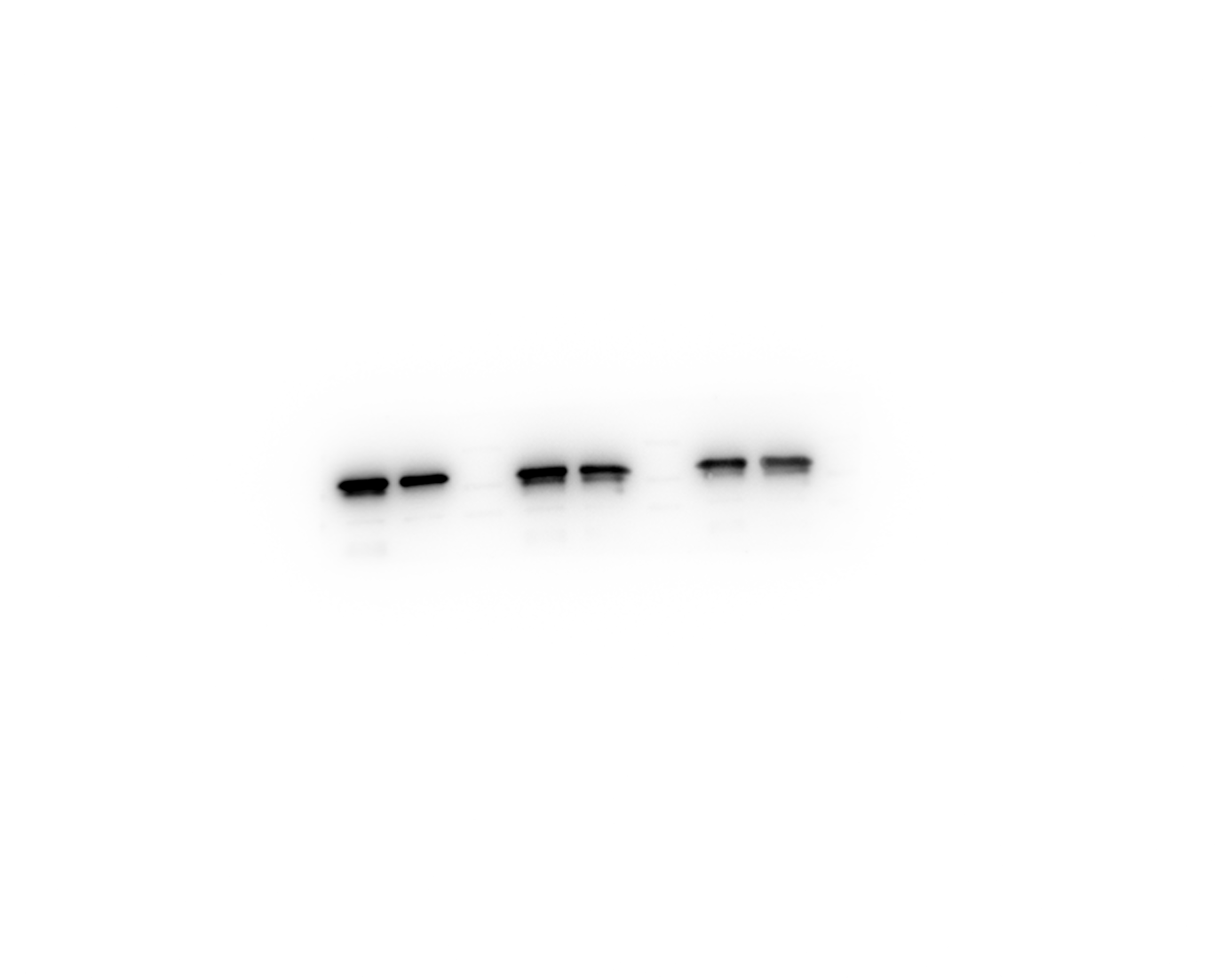

Supplement: Figure 9—figure supplement 1—source data 2. [file elife-107104-fig9-figsupp1-data2.zip › Figure 9-figure supplement 1-source data 2. Original files for western blot analysis/Figure 9-figure supplement 1A-3.tif]

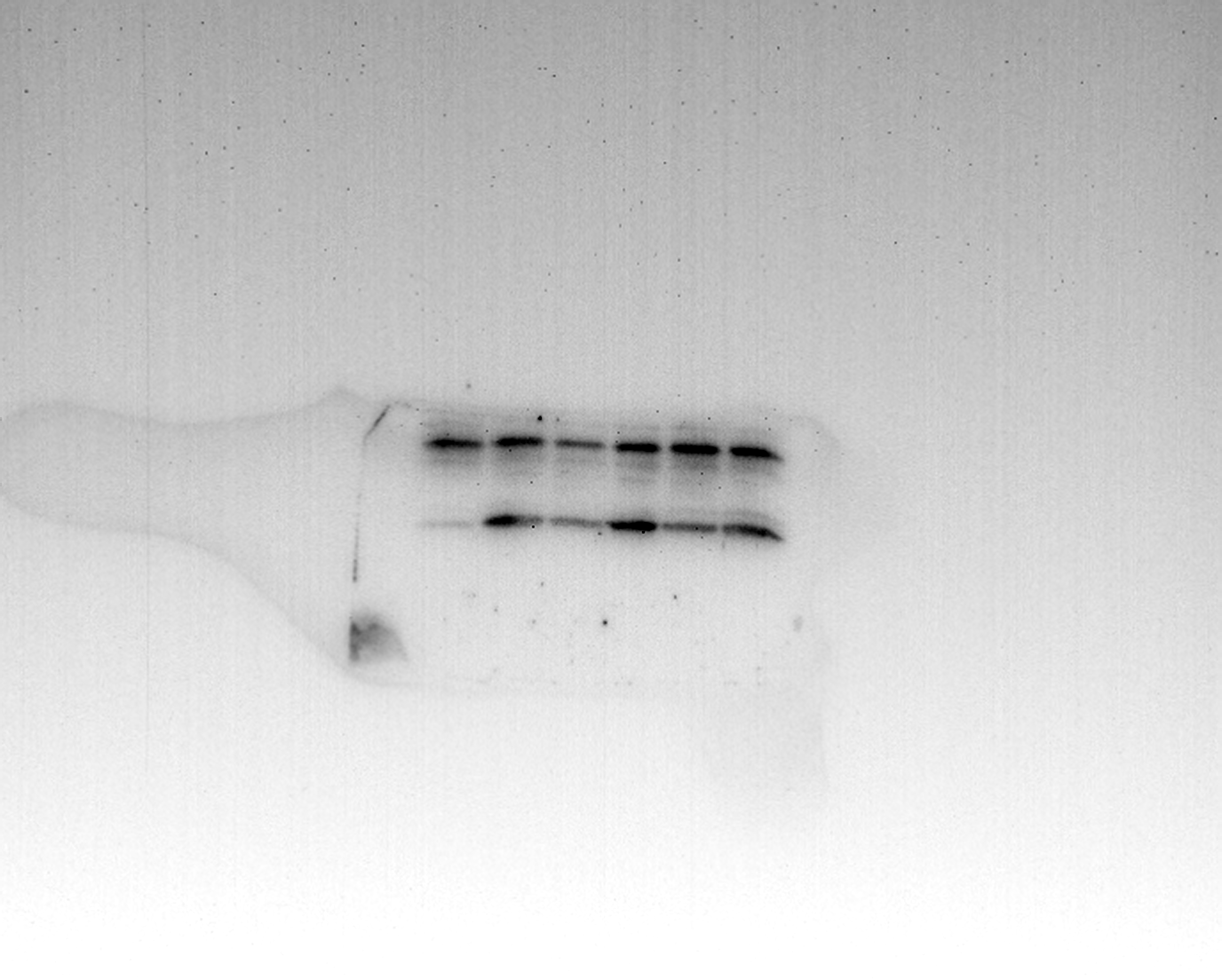

Supplement: Figure 9—figure supplement 1—source data 2. [file elife-107104-fig9-figsupp1-data2.zip › Figure 9-figure supplement 1-source data 2. Original files for western blot analysis/Figure 9-figure supplement 1B-1.tif]

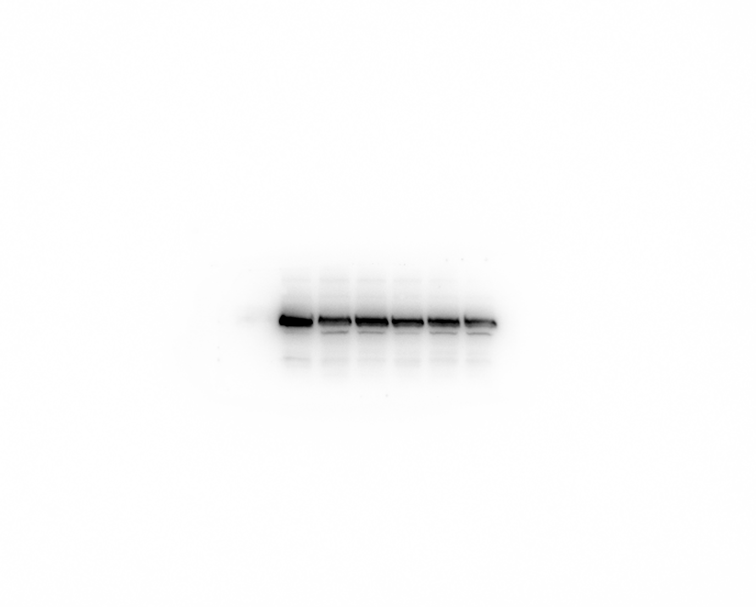

Supplement: Figure 9—figure supplement 1—source data 2. [file elife-107104-fig9-figsupp1-data2.zip › Figure 9-figure supplement 1-source data 2. Original files for western blot analysis/Figure 9-figure supplement 1B-2.tif]

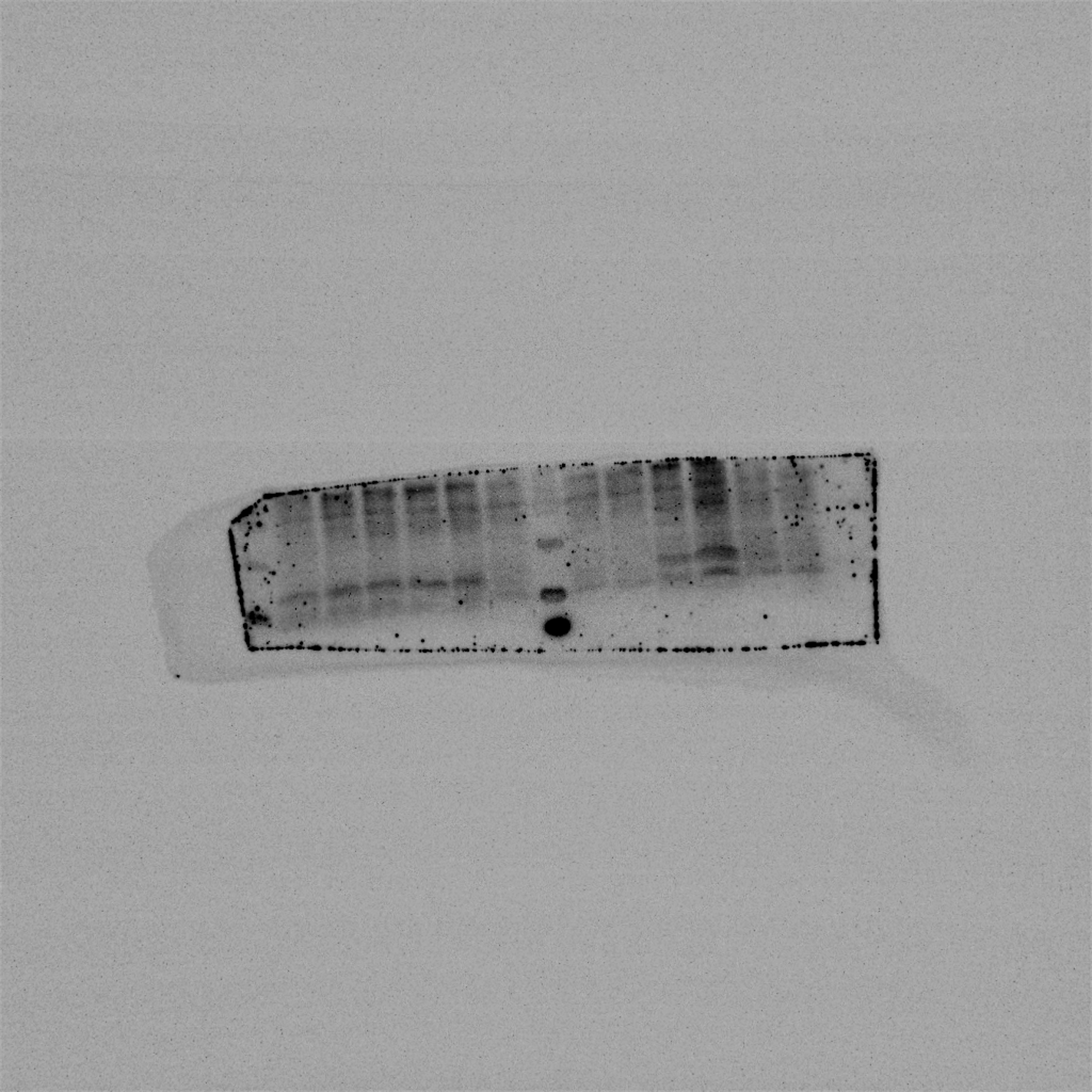

Supplement: Figure 9—figure supplement 1—source data 2. [file elife-107104-fig9-figsupp1-data2.zip › Figure 9-figure supplement 1-source data 2. Original files for western blot analysis/Figure 9-figure supplement 1B-3.tif]

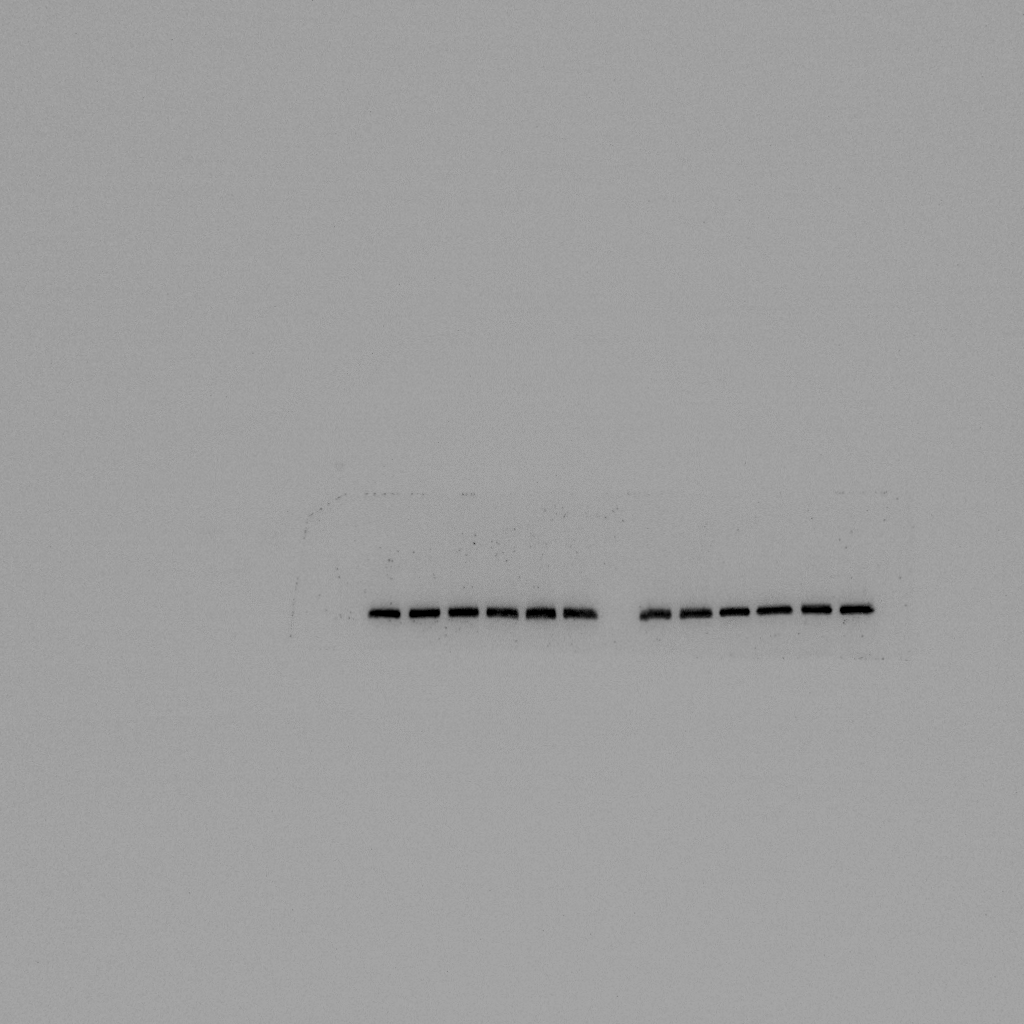

Supplement: Figure 9—figure supplement 1—source data 2. [file elife-107104-fig9-figsupp1-data2.zip › Figure 9-figure supplement 1-source data 2. Original files for western blot analysis/Figure 9-figure supplement 1B-4.tif]
